# Calmodulin-like protein 3 is an estrogen receptor alpha coregulator for gene expression and drug response in a SNP, estrogen, and SERM-dependent fashion

**DOI:** 10.1186/s13058-017-0890-x

**Published:** 2017-08-18

**Authors:** Sisi Qin, James N. Ingle, Mohan Liu, Jia Yu, D. Lawrence Wickerham, Michiaki Kubo, Richard M. Weinshilboum, Liewei Wang

**Affiliations:** 1 Department of Molecular Pharmacology and Experimental Therapeutics, Mayo Clinic, Rochester, MN USA; 2 Department of Medical Oncology, Mayo Clinic, Rochester, MN USA; 3 Section of Cancer Genetics and Prevention, Allegheny Health Network Cancer Institute, Pittsburgh, PA USA; 4 National Surgical Adjuvant Breast and Bowel Project (NRG Oncology), Pittsburgh, PA USA; 5 Laboratory for Genotyping Development, Center for Genomic Medicine, RIKEN, Yokohama, Japan

**Keywords:** ERα coregulator, Single nucleotide polymorphism, Estrogen, Selective estrogen receptor modulator treatment

## Abstract

**Background:**

We previously performed a case–control genome-wide association study in women treated with selective estrogen receptor modulators (SERMs) for breast cancer prevention and identified single nucleotide polymorphisms (SNPs) in *ZNF423* as potential biomarkers for response to SERM therapy. The *ZNF423*rs9940645 SNP, which is approximately 200 bp away from the estrogen response elements, resulted in the SNP, estrogen, and SERM-dependent regulation of ZNF423 expression and, “downstream”, that of BRCA1.

**Methods:**

Electrophoretic mobility shift assay–mass spectrometry was performed to identify proteins binding to the *ZNF423* SNP and coordinating with estrogen receptor alpha (ERα). Clustered, regularly interspaced short palindromic repeats (CRISPR)/Cas9 genome editing was applied to generate ZR75-1 breast cancer cells with different *ZNF423* SNP genotypes. Both cultured cells and mouse xenograft models with different *ZNF423* SNP genotypes were used to study the cellular responses to SERMs and poly(ADP-ribose) polymerase (PARP) inhibitors.

**Results:**

We identified calmodulin-like protein 3 (CALML3) as a key sensor of this SNP and a coregulator of ERα, which contributes to differential gene transcription regulation in an estrogen and SERM-dependent fashion. Furthermore, using CRISPR/Cas9-engineered ZR75-1 breast cancer cells with different *ZNF423* SNP genotypes, striking differences in cellular responses to SERMs and PARP inhibitors, alone or in combination, were observed not only in cells but also in a mouse xenograft model.

**Conclusions:**

Our results have demonstrated the mechanism by which the *ZNF423* rs9940645 SNP might regulate gene expression and drug response as well as its potential role in achieving more highly individualized breast cancer therapy.

**Electronic supplementary material:**

The online version of this article (doi:10.1186/s13058-017-0890-x) contains supplementary material, which is available to authorized users.

## Background

Breast cancer is the most common invasive cancer of women worldwide [1]. The selective estrogen receptor modulators (SERMs) tamoxifen and raloxifene, drugs that compete with estrogens for binding to the estrogen receptor (ER), have been approved by the US Food and Drug Administration for breast cancer prevention in high-risk women. The largest and most influential breast cancer chemoprevention trials were the double-blind, placebo-controlled National Surgical Adjuvant Breast and Bowel Project (NSABP) P-1 trial of tamoxifen [2] and the double-blind NSABP P-2 trial that compared raloxifene with tamoxifen [3, 4]. Involving over 33,000 women, these two trials showed that tamoxifen and raloxifene reduced the occurrence of breast cancer in women at high risk by approximately half. However, SERM prevention has not been widely adopted clinically [5, 6], in part because of rare but serious side effects [7] and also because of the large number of women who must be treated to prevent one case of breast cancer. It would be a major advance for breast cancer prevention if we could identify and understand biomarkers that could help to identify the women most likely to benefit from SERM treatment.

Our previous discovery genome-wide association study (GWAS) using samples from NSABP P-1 and P-2 subjects identified common single nucleotide polymorphisms (SNPs) in the *ZNF423* gene as potential biomarkers for individualized SERM prevention therapy [8]. One of those SNPs, rs9940645 located approximately 200 bp distant from several estrogen response elements (EREs), resulted in SNP, estrogen and SERM-dependent regulation of ZNF423 expression and, “downstream”, that of BRCA1. Specifically, we found increased expression of ZNF423 and BRCA1 in the presence of E2 but decreased expression when 4-hydroxytamoxifen (4-OH-TAM) was present for the WT SNP genotype. The opposite regulations of ZNF423 and BRCA1 expression was observed when treated with either E2 or 4-OH-TAM for the variant SNP. Although ZNF423 functions as a DNA-binding transcription factor in several signaling pathways [9, 10], its role in breast cancer and treatment response remains unknown. We have shown that ZNF423 directly regulated BRCA1 expression and influenced its function in DNA damage repair [8]. Therefore, the SNP and the level of ZNF423 expression might also have a significant effect on response to the poly(ADP-ribose) polymerase (PARP) inhibitors that have shown significant therapeutic effect in patients with BRCA1/2 deficiency [11–13]. It is possible that the rs9940645 SNP in the *ZNF423* gene might be used as a biomarker to select patients for therapy with PARP inhibitors, either alone or in combination with SERMs, especially in patients who have low BRCA1 expression resulting from the effect of *ZNF423* SNP genotypes in the presence of different drug treatments.

In the present study, we demonstrated how the *ZNF423* rs9940645 SNP that was not within an ERE was able to affect the expression of ZNF423 and BRCA1 as well as treatment response as a result of the actions of calmodulin-like protein 3 (CALML3), which we identified as part of a complex bound to the *ZNF423* SNP. CALML3 is a calcium-sensing protein known to be highly expressed in epithelial cells in tissues like breast, prostate and skin [14, 15]. Previous work has shown that it is a regulator of myosin-10 [16, 17], which may be important in cell adhesion and motility [18–20]. CALML3 is downregulated in breast cancer and transformed cells in culture [15, 21]. However, no prior information is available with regard to its role in transcription regulation. Our study indicated that CALML3 functions as a “sensor” for different SNP genotypes and that, together with ERα, it regulates ZNF423 expression and, in turn, BRCA1 expression in a SNP, estrogen and SERM-dependent fashion. We then performed studies in ERα + breast cancer cells selected on the basis of *ZNF423* SNP genotypes, and confirmed those results in clustered, regularly interspaced short palindromic repeats (CRISPR)-engineered ZR75-1 breast cancer cells with different *ZNF423* SNP genotypes. Finally, we investigated the SNP effect on response to a series of anti-neoplastic drugs including PARP inhibitors, either alone or in combination with SERMs.

## Methods

### CRISPR/Cas9 genome editing

To change the *ZNF423* rs9940645 SNP from variant to WT in ZR75-1 cells which had the variant sequence at that location, we purchased custom-designed CasGuide and Donor vectors from Blue Heron Biotech (An Origene Company for Gene Synthesis, Bothell, WA, USA). Because we wanted to change only a single nucleotide, no selection tag was introduced into the genome.

Specifically, ZR75-1 breast cancer cells, which are ERα + and carry the *ZNF423* variant SNP, were cotransfected with pCasGuide and pUCminusMCS Donor DNA (with the WT SNP sequence) according to lipofectamine3000 (Life Technologies, Gaithersburg, MD, USA) instructions. After 48 hours, cells were split 1:10, grown for an additional 3 days, and then split 1:10 again. After 10 days, DNA was isolated from the transfected cells in these 100 wells and the genotypes of the cells in each well were determined by TaqMan SNP Genotyping Assays (Thermo Fisher Scientific, Waltham, MA, USA) for *ZNF423* rs9940645. Cells with a higher ratio of WT to variant allele values were selected and monoclones were generated. Approximately 3 months later, cells grown from the monoclones were again screened by TaqMan SNP Genotyping Assays and the DNA sequences of selected clones carrying the WT SNP were validated by Sanger sequencing. This procedure allowed us to obtain a CRISPR-ZR75-1 cell line that was homozygous for the *ZNF423* WT SNP genotype.

### Cell culture, transfection and drug treatment

The human ERα + breast cancer cell lines T47D, BT474, ZR75-1 and CAMA-1 were obtained from the American Type Culture Collection (ATCC, Manassas, VA, USA). Hs578T-ERα, which was stably transfected with ERα, was a generous gift from Thomas Spelsberg, PhD (Mayo Clinic, Rochester, MN, USA). T47D, BT474 and ZR75-1 cells were cultured in RPMI 1640 medium (Gibco, Grand Island, NY, USA), CAMA-1 cells were cultured in Eagle's Minimum Essential Medium (EMEM) (ATCC), and Hs578T-ERα cells were cultured in Dulbecco’s Modified Eagle Medium (DMEM) (Gibco). All of the media were supplemented with 10% fetal bovine serum (FBS) (Atlanta Biologicals, GA, USA).

Lymphoblastoid cell lines (LCLs) with known genotypes for the *ZNF423* SNP were obtained from the Coriell Cell Repository (Camden, NJ, USA) and were cultured in RPMI 1640 medium containing 15% FBS (Atlanta Biologicals). Pre-overexpressed ERα LCLs were described previously [8].

GM17239, GM17272 and GM17252 with the *ZNF423* variant SNP genotype and GM17214, GM17282 and GM17285 with the *ZNF423* WT SNP genotype were used for electrophoretic mobility shift assay (EMSA) and drug cytotoxicity after knockdown of ZNF423 and CALML3. Additional WT LCLs GM17257, GM17281 and GM17278 and variant cell lines GM17236, GM17296 and GM17294 were used for drug combination cytotoxicity assays.

Prior to transfection and estradiol (E2) treatment, cells were grown in phenol red-free media containing 5% charcoal-stripped serum (Thermo Fisher Scientific, Waltham, MA, USA) for 48 hours. The cells were then reverse-transfected with two different short interfering RNAs (siRNAs) for target genes (ZNF423, D-012907-01 and D-012907-03; CALML3, D-019122-01 and D-019122-02; ESR1, D-003401-01 and D-003401-02) or with negative control siRNA (D-001206-13; Dharmacon, Lafayette, CO, USA) using the Lipofectamine RNAiMAX Reagent (Thermo Fisher Scientific) according to the manufacturer’s instructions. After 24 hours, cells were incubated with 0.01 nM E2 for an additional 24 hours (Sigma-Aldrich, St. Louis, MO, USA), followed by the addition of 10^–7^ μM 4-hydroxytamoxifene (4-OH-TAM) (Sigma-Aldrich) or 10^–7^μM raloxifene (Sigma-Aldrich). To mimic calcium-reduced or free conditions, a selective intracellular membrane-permeable calcium chelator, BAPTA-AM (Sigma-Aldrich) at a concentration of 0.5 or 5 μM, was added to the media 24 hours after E2 treatment. As vehicle controls, ethanol for E2 and DMSO for 4-OH-TAM, raloxifene and BAPTA-AM were added to the medium at a final concentration < 0.1%. Cells were collected 72 hours after transfection.

### EMSA and mass spectrometry

Selected LCLs treated with different drugs were washed with PBS and nuclear proteins were extracted in cell lysis buffer (10 mM HEPES; pH 7.5, 10 mM KCl, 0.1 mM EDTA, 1 mM dithiothreitol (DTT), 0.5% Nonidet‐40) with a protease and phosphatase inhibitor cocktail (Roche, Basel, Switzerland). Then 20-μl DNA–protein mixtures were prepared by adding 1 nM biotin-labeled DNA oligonucleotide probes containing either wild-type (WT) or variant *ZNF423* SNP genotypes into various quantities of protein in 1× binding buffer (1 mM Tris, 6 mM NaCl, 0.5 mM MgCl_2_, 0.01 mM EDTA, 0.1 mM CaCl_2_, 0.2% glycerol). These mixtures were incubated for 30 min at RT. Unlabeled DNA probes containing the *ZNF423* rs9940645 SNP (TGACTGTCATAT**T/C**GTGGCTTTTCTG) were added for the competitive binding assays. The DNA–protein complexes were then separated on 6% native polyacrylamide gel using Tris Borate EDTA [6] at 4 °C.

**Fig. 1:**
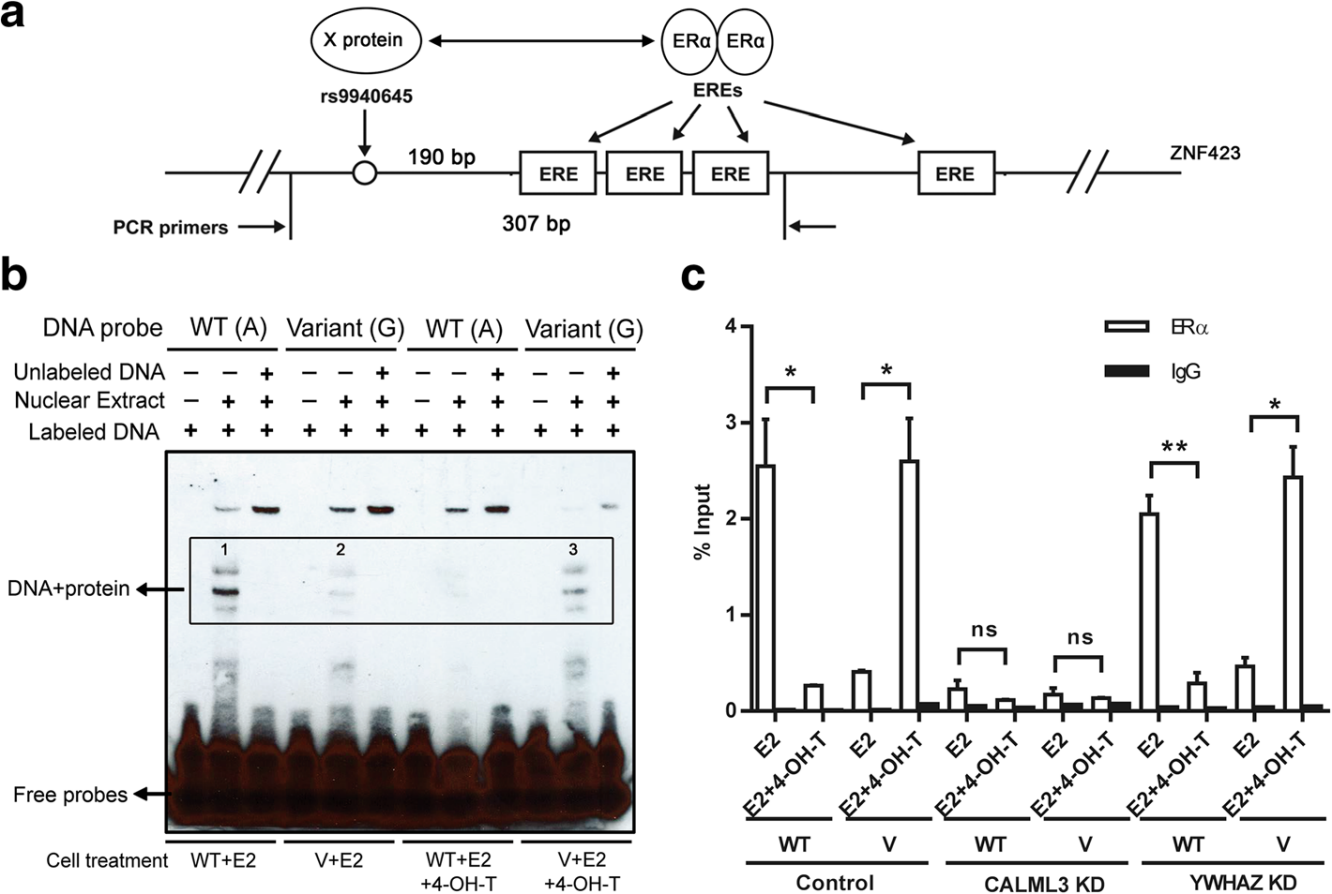
CALML3 as a potential ERα coregulator binds to the *ZNF423* rs9940645 SNP. **a** Schematic representation of the genomic region surrounding the rs9940645 SNP (in intron 1) in the *ZNF423* gene. The SNP is in intron 2 of *ZNF423* and 240 bp away from the closest ERE. **b** EMSA to identify the candidate proteins that bind differentially to WT and variant SNPs sequences in LCLs treated with E2 ± 4-OH-TAM. *1*–*3* bands indicating specific DNA–protein binding. **c** Further validation of CALML3 as a candidate protein by ChIP-qPCR using ERα antibody after the knockdown of CALML3 in WT and variant LCLs treated with E2 ± 4-OH-TAM. Primers used for amplification are indicated in (**a**) with *arrows*, so the amplicons included both the SNP and EREs. YWHAZ as a nonspecific SNP-binding protein identified by MS was also included as a negative control. Enrichment of DNA fragments shown as the mean (± SEM) of three independent experiments. *p < 0.05, **p < 0.01 by two-tailed Student’s *t* test. *4-OH-TAM* 4-hydroxytamoxifene, *E2* estradiol, *ER*α estrogen receptor alpha, *ERE* estrogen response element, *ns* not significant, *WT* wild type

After electrophoresis, the gels were silver stained (Thermo scientific kits) and all three bands (indicated as 1, 2 and 3 in Fig. 1b) indicating specific DNA–protein binding were cut from the gels and were sent for mass spectrometry (MS). Proteins that were identified in all three bands were selected as candidates. Western blot analysis was performed to validate which proteins bound to DNA oligonucleotide probes containing the *ZNF423* SNP.

### Real-time quantitative reverse transcription-PCR

Total RNA was isolated from treated cells with the Qiagen RNeasy kit (QIAGEN, Hilden, Germany), and 100 ng of total RNA was used to perform quantitative reverse transcription-PCR (qRT-PCR) using primers for Beta-ACTIN, ESR1, ZNF423, CALML3, BRCA1 and YWHAZ (QIAGEN) with Power SYBR Green RT-PCR Reagents (Life Technologies, Carlsbad, CA, USA). All experiments were performed in triplicate with Beta-ACTIN (QIAGEN) as an internal control.

### Coimmunoprecipitation and antibodies

Proteins from treated cells were extracted using NETN buffer (100 mM NaCl, 20 mM Tris–Cl (pH 8.0), 0.5 mM EDTA, and 0.5% Nonidet P-40) with protease and phosphatase inhibitor cocktails (Roche). Coimmunoprecipitation (Co-IP) was then performed as described previously [22]. Antibodies against Beta-Actin (A1978; Sigma-Aldrich), ERα (MA3-310; Thermo Fisher Scientific), ZNF423 (Ab189608; Abcam, Cambridge, UK), CALML3 (Ab118689; Abcam) and BRCA1 (sc-135731; Santa Cruz biotechnology, Dallas, TX, USA) were used to perform the Co-IP and western blot analysis.

### Chromatin immunoprecipitation

Chromatin immunoprecipitation (ChIP) [23] was performed using the EpiTect ChIP one-day kit (QIAGEN). Quantitative PCR was used to monitor ChIP results with RT^2^ SYBR Green PCR Master Mix reagent (Life Technologies). For the ChIP assay designed to determine protein complex binding at or near the *ZNF423* rs9940645 SNP, the primer sequences were indicated as follows: primers used to amplify the region containing SNP and ERE, 5′-GTTCTCTTTTCAGCAAGTGGTCA-3′ and 5′-ATCATCTTTGGCATCACGCTC-3′; primers used to amplify the region only containing the SNP, 5′-GTTCTCTTTTCAGCAAGTGGTCA-3′ and 5′-GTGCAGACAAACCCAACGAGG-3′; primers used to amplify the region containing all four EREs (nearby and distal), 5′-CTCGTTGGGTTTGTCTGCAC-3′ and 5′-GTGGCGAGAAAGAGCTGAAA-3′; primers used to amplify the region containing the three nearby EREs, 5′-CTCGTTGGGTTTGTCTGCAC-3′ and 5′-ATCATCTTTGGCATCACGCTC-3′; and primers used to amplify the region only containing the distal ERE, 5′-GAGCGTGATGCCAAAGATGAT-3′ and 5′-GTGGCGAGAAAGAGCTGAAA-3′.

### Cytotoxicity assays

Cytotoxicity assays were performed in triplicate for cells treated with increasing concentrations of drug for 3 days, followed by CellTiter 96 AQueous Non-Radioactive Cell Proliferation Assays (Promega, Madison, WI, USA) which were performed as described previously [24].

### Colony-forming assays

After reverse transfection of *ZNF423* or *CALML3* siRNAs, approximately 500–1000 cells were seeded in six-well plates and were treated with 0.01 nM E2 combined with 4-OH-TAM, raloxifene, olaparib or cisplatin (Sigma-Aldrich). IC50 values were determined by cytotoxicity assays. Media and drugs were replenished every 3 days. After 14–28 days, cells were stained with crystal violet and the number of clones was counted. All experiments were performed in triplicate. For rescue experiments, cells with downregulated ZNF423 or CALML3 were overexpressed with a BRCA1 construct prior to drug treatment.

### Statistical analysis

Data were analyzed using GraphPad Prism Software. Student’s two-tailed *t* test was used for comparison of relative mRNA expression levels by qRT-PCR, DNA fragment enrichment in ChIP assays, clone numbers in colony formation and tumor volume in the xenograft model. *p* < 0.05 was considered statistically significant.

### Animal studies

The animal studies were reviewed and approved by the Mayo Institutional Animal Care and Use Committee (IACUC). Breast cancer xenografts generated from ZR75-1 cells with *ZNF423* variant and CRISPR-engineered WT SNP genotypes were used to test the tumor response to treatment with a PARP inhibitor ± tamoxifen. Six-week-old female athymic nu/nu mice were water-fed with a low dose of E2 (70 μg) every week. Then 2 × 10^6^ logarithmically growing breast cancer cell lines were mixed 1:1 with growth-factor reduced, phenol red-free matrigel (BD-Diagnostic System, Franklin Lakes, NJ, USA). The cell mixture was diluted in 100 μl PBS and injected subcutaneously into mice using an 18-G needle under the skin overlying the sacroiliac joint. Tumor growth was monitored daily until tumors reach an appropriate size of 100 mm^3^. The mice were then randomized into groups treated with PBS as control, tamoxifen (5 mg/kg/day), PARP inhibitor (olaparib, 40 mg/kg/day) alone or PARP inhibitor plus tamoxifen for 30 days. Tamoxifen citrate, olaparib or saline controls containing 10% (w/v) 2-hydroxy-propyl-betacyclodextrin (Sigma) were injected intraperitoneally into the mice every morning in a volume of 0.2 ml. Mice weight and tumor growth were monitored every 3 days by measuring the tumor length (*L*) and width (*W*) using a caliper, and the tumor volume [25] was calculated using the formula:

$$ \mathrm{TV}=\left(L\times {W}^2\right)/2. $$

When tumors in the control mice reached a size at which the mice had to be sacrificed, the tumors were removed and saved for further analysis.

## Results

### CALML3 binds to the *ZNF423* rs9940645 SNP and regulates the SNP-dependent binding of ERα to EREs

We reported previously that the rs9940645 SNP in *ZNF423* was associated with SERM response in the NSABP P-1 and P-2 trials [8]. The genotype for that SNP was also found to affect the expression of ZNF423 and BRCA1 in response to estrogen and SERM exposure. Because this SNP was not in an ERE but 200 bp away (Fig. 1a), we hypothesized that there might be ERα coregulators binding directly to the DNA region containing the SNP and “coordinating” with ERα to regulate ZNF423 expression in a SNP-dependent fashion.

As a first step to test this hypothesis, we designed DNA probes containing either the WT or variant rs9940645 SNP genotype sequences and performed electrophoretic mobility shift assays (EMSAs) using nuclear extracts from lymphoblastoid cell lines (LCLs) selected based on different *ZNF423* SNP genotypes that had been treated with E2 alone or E2 plus 4-OH-TAM, the active metabolite of tamoxifen [8] (Fig. 1a). This cell line system had been used to validate the SNP and drug-dependent effect on *ZNF423* and *BRCA1* gene expression in our previous studies [8]. After EMSA, four bands indicating specific DNA–protein interaction showed different intensities based on genotypes in the presence of different treatments, indicating that the affinity of proteins binding to the DNA probe was different for different SNP genotypes. However, one band using nuclear extracts from WT LCLs treated with E2 plus 4-OH-TAM was very weak (Fig. 1b). Therefore, we isolated the three bands with three distinct shifts that contained DNA–protein complex (Fig. 1a) and that could be clearly observed on the gel based on their shifts, and sent them for mass spectrometry (MS) protein identification (Fig. 1b) [6]. As the patterns of shifts were very similar across different treatments, a list of common proteins identified by MS in all three bands but bound differentially to WT and variant *ZNF423* SNPs were obtained based on a protein match score > 10 (Additional file 1). Included among these proteins were ACTA2, ACTB, ACTBL2, ANXA2P2, CALML3, HSP90AA1, LMNA, MYH9, TUBA1C and YWHAZ (see Additional file 1 for MS results for the three bands individually and a Venn diagram for MS summary). To validate the MS results, we performed western blot analysis by incubating specific antibodies with the EMSA membrane. We eliminated proteins that were commonly seen and that might be nonspecific binding proteins in the MS analysis due to their abundance in cells, such as ACTB, LMNA, HSP90AA1 and MYH9 [25, 26]. We then selected the following proteins for experimental testing: ACTA2, ACTBL2, ANXA2P2, CALML3, TUBA1C and YWHAZ. Among this group of proteins, only CALML3 showed a positive signal in all three bands (Additional file 2: Figure S1). The reference list of nonspecific proteins was identified based on the MS of IP products [25, 26], which is different from the proteins we identified here that are in the DNA–protein complexes from the EMSA experiments. Therefore, we also experimentally validated some of the nonspecific proteins including ACTB, LMNA, HSP90AA1 and MYH9. Our results showed that among all of the proteins we tested, including potential specific and selected nonspecific proteins, only CALML3 showed a functional effect.

To first determine whether CALML3 might alter ERα binding to the genomic region containing both the SNP as well as the nearby EREs, CALML3 was knocked down in both WT and variant LCLs treated with E2 alone or E2 plus 4-OH-TAM, and then ChIP assays were performed with ERα antibody (Fig. 1c). The control group across different treatments showed a striking SNP-dependent ERα binding pattern, with more ERα protein binding to the region containing the WT sequence in the presence of E2, whereas much more ERα protein binding was observed for the variant SNP genotype when 4-OH-TAM was added. Knockdown of CALML3 abolished the SNP and drug-dependent differential ERα binding. In addition, knockdown of YWHAZ, one of the candidates that cannot be verified by the western blot analysis as our negative control, did not change the binding (Fig. 1c). These results suggested that CALML3, together with ERα, might be involved in the *ZNF423* SNP-dependent gene regulation.

### CALML3 functions as a sensor of the *ZNF423* rs9940645 SNP and regulates ZNF423 expression with ERα in ZR75-1 WT and variant cells

**Fig. 2:**
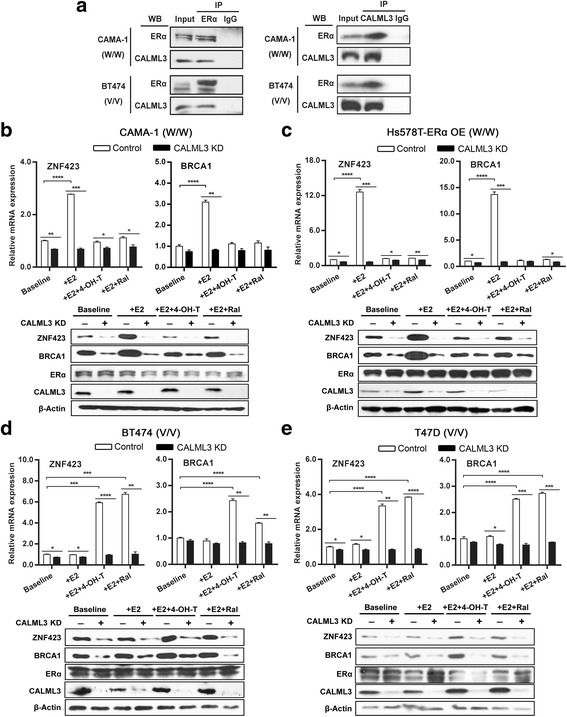
CALML3 as a coregulator of ERα affects the expression of ZNF423 and BRCA1. **a** Interaction of ERα and CALML3 in *ZNF423* rs9940645 WT or variant genotype breast cancer cells treated with 0.01 nM E2. Co-IP performed by immunoprecipitating the protein complex with anti-ERα antibody, followed by immunoblotting for CALML3, or vice versa. **b**–**e** CALML3 was knocked down in ERα + breast cancer cells homozygous for *ZNF423* WT or variant SNP genotypes and followed by treatment with 0.01 nM E2 ± 4-OH-TAM/raloxifene 10^–7^ mol/L. ZNF423 and BRCA1mRNA expression levels relative to beta-ACTN shown as the mean of three independent experiments (± SEM). *p < 0.05, **p < 0.01, ***p < 0.001, ****p < 0.0001 by two-tailed Student’s *t* test. Protein levels determined by western blot (*WB*) analysis. *IP* immunoprecipitation, *4-OH-TAM* 4-hydroxytamoxifene, *E2* estradiol, *ER*α estrogen receptor alpha, *Ral* raloxifene

CALML3 is a calcium-sensing protein known to be highly expressed in epithelial cells, including tissues such as breast [14, 15], and it is downregulated in breast cancers and transformed cells in culture [15, 21]. However, the mechanism by which CALML3 might contribute to the regulation of ZNF423 and BRCA1 expression is not known. Based on the observations we made in LCLs, we asked whether CALML3 might function as an ERα regulator, cooperating with ERα to regulate ZNF423 expression in ERα + breast cancer cells. As a first step, we performed immunoprecipitation to confirm the endogenous interaction between CALML3 and ERα in both CAMA-1 cells containing the *ZNF423* WT genotype and BT474 cells containing the *ZNF423* variant genotype in the presence of E2 (Fig. 2a). There is no interaction without E2 (data not shown).

Next we asked whether the SNP effect is also present in breast cancer cells because LCLs obviously have gene expression profiles differing from those of breast cancer cells. We then selected a panel of breast cancer cells on the basis of *ZNF423* SNP genotypes, specifically two cell lines containing the WT genotype and two containing the variant genotype to further confirm the SNP-dependent effect. We knocked down CALML3 and ERα individually in these breast cancer cells that had been treated with E2 alone or E2 in combination with SERMs, followed by determining mRNA and protein levels. Observations very similar to those made in LCLs with regard to the SNP-dependent effects were also seen in these cancer cells. Specifically, cells with the WT *ZNF423* rs9940645 SNP displayed a more than 2-fold change of ZNF423 and BRCA1 mRNA in the presence of 0.01 nM E2 (Fig. 2b, c, white bars), whereas ZNF423 and BRCA1 expression was not significantly altered in cells containing the variant SNP genotype (Fig. 2d, e, white bars). However, when SERMs were added in addition to E2, expression of both genes was increased in cells with the variant genotype (Fig. 2d, e, white bars). When compared to the controls (negsiRNA), knockdown of CALML3 abolished the SNP-dependent regulation of ZNF423 and BRAC1 in cells treated with E2 or E2 plus 4-OH-TAM, or raloxifene (Fig. 2b–e, black bars). Protein levels were consistent with the mRNA expression results (Fig. 2b–e, lower panel). Using a second CALML3 siRNA showed the same results (Additional file 2: Figure S2). On the other hand, although knocking down ERα decreased the expression of ZNF423 and BRCA1, it did not abolish SNP and drug-dependent effects, suggesting that ERα activated the transcription of ZNF423 but was not the sensor for the effect of different SNP genotypes (Additional file 2: Figure S3A–D, black bars).

**Fig. 3:**
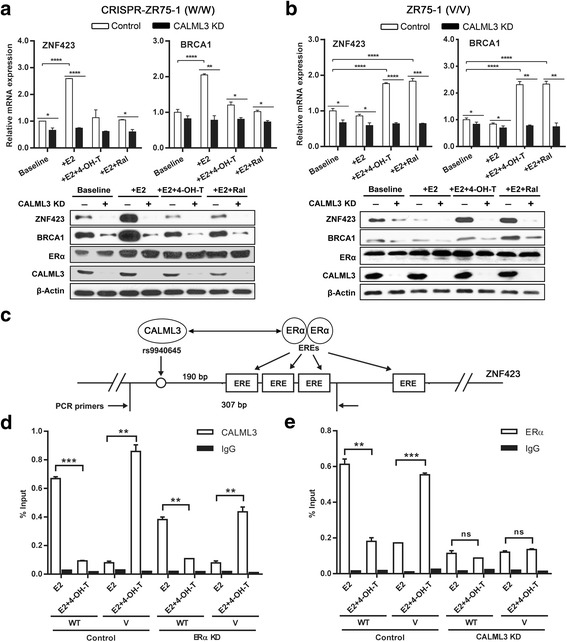
Effect of SNP-dependent regulation of CALML3 and ERα on the expression of ZNF423. **a**, **b** Expression of ZNF423 and BRCA1 determined by qRT-PCR after knocking down CALML3 in ERα + ZR75-1 breast cancer cells with *ZNF423* WT or variant genotypes. mRNA expression levels shown as the mean of three independent experiments (± SEM), comparisons made by two-tailed Student’s *t* test. Protein levels were determined by western blot analysis. **c** Schematic representation shows the role of CALML3 in the SNP-dependent effect of the ER dimer. **d**, **e** ChIP-qPCR assays performed using CALML3 antibody after knocking down ERα (**d**) or vice versa (**e**) in CRISPR-ZR75-1 cells (WT) and ZR75-1 cells (variant) treated with E2 ± 4-OH-TAM/raloxifen. Primer locations indicated in (**c**). Enrichment of DNA fragments (three independent experiments, mean ± SEM) between different treatments was compared by two-tailed Student’s *t* test. *p < 0.05, **p < 0.01, ***p < 0.001, ****p < 0.0001. *4-OH-TAM* 4-hydroxytamoxifene, *E2* estradiol, *ER*α estrogen receptor alpha, *ERE* estrogen response element, *Ral* raloxifene, *V* variant, *WT* wild type

To further determine whether the differential regulation of gene expression in the presence of E2 ± SERMs was due entirely to the SNP effect rather than the heterogeneity of the cancer cells, the ZR75-1 cell line, an ERα + breast cancer cell line that naturally contains the *ZNF423* variant SNP genotype, was CRISPR engineered to form the WT sequence. This paired cell line provided us with a cell line model system with an isogenic background to study only the single SNP effect. In these paired cell lines with different *ZNF423* rs9940645SNP genotypes, we validated the results observed in LCLs and breast cancer cells in that knocking down CALML3 abolished the SNP-dependent gene regulation of ZNF423 and BRCA1 expression in cells treated with E2 ± SERMs (Fig. 3a, b; Additional file 2: Figure S3E, F).The binding of CALML3 and ERα to the genomic region containing the SNP in the presence of E2 ± SERM treatment was also investigated. ChIP assays using anti-CALML3 antibody were performed for control cells and cells with ERα knockdown that had been treated with E2 alone or E2 plus 4-OH-TAM (Fig. 3d). ChIP assays using anti-ERα antibody were also performed under the same conditions (Fig. 3e). Our ChIP assays included the region containing both the SNP and the nearby EREs that is approximately 200 bp away from the SNP (Fig. 3c). In the control groups, without gene knockdown, CALML3 and ERα both showed differential binding to the genomic region containing either WT or variant SNP sequences (Fig. 3d, e). The results were consistent with the differential ZNF423 and BRCA1 expression described earlier. However, knocking down ERα resulted in a decreased binding of CALML3 to the region containing the SNP and the nearest EREs from the SNP, but did not change the differential binding pattern between the WT and the variant (Fig. 3d). However, knocking down CALML3 not only decreased ERα binding to the region containing the SNP+ nearby EREs, but also abolished the SNP-dependent DNA–protein binding pattern and, in turn, the SNP-dependent ZNF423 expression pattern (Fig. 3e). The binding of ERα is dependent on E2 because we did not see any binding of ERα to the DNA region without E2 (data not shown). There is another ERE which we called the distal ERE relative to the one described earlier. This distal ERE is located 345 bp away from the SNP (Additional file 2: Figure S4A). We also repeated the ChIP assays with PCR amplicons containing only the SNP (Additional file 2: Figure S4B), all EREs, only three nearby EREs or the distal ERE (Additional file 2: Figure S4C–E). Based on those PCR results, ERα showed little binding to only the SNP region (Additional file 2: Figure S4B) and CALML3 showed little binding to the region containing all of the EREs (three nearby plus the distal ERE) (Additional file 2: Figure S4C). The binding of ERα to only the three nearby EREs was very similar to the distal ERE, while the binding of ERα to the distal ERE is much lower, with little difference between different treatments (Additional file 2: Figure S4D, E). In addition, knockdown of CALML3 had little effect on the binding pattern of ERα to the distal ERE in different genotypes or treatment background, suggesting that this distal ERE is not involved in SNP and drug-dependent CALML3 regulation of ZNF423 transcription. These results further suggest that ZNF423 transcription is dependent on the ERα binding to the nearby EREs that are 200 bp away from the SNP and that CALML3 is the key sensor of the *ZNF423* rs9940645 SNP which is involved in the SNP-dependent regulation of ZNF423.

As a member of the calmodulin family, CALML3 is capable of binding calcium, acting as a signal transducer [14, 15]. We then tested whether its regulation of ZNF423 is calcium dependent by reducing the free intracellular calcium level using a selective calcium chelator BAPTA-AM. Our results showed that CALML3 interaction with ERα was calcium dependent (Additional file 2: Figure S5A), but its binding to the *ZNF423* SNP sequence was independent of calcium (Additional file 2: Figure S5B). To further test whether CALML3 has a broader effect on ER function, we selected some canonical ER-targeted genes and determined their gene expression after knocking down CALML3. The mRNA expression of those canonical ER-targeted genes including EFP, COX7RP and EBAG9 were induced after E2 treatment as expected in breast cancer cells, but did not change significantly after knockdown of CALML3 compared to the control (Additional file 2: Figure S5C).

### CALML3 and ZNF423 influence cellular response to SERMs, PARP inhibitor and platinum

**Fig. 4:**
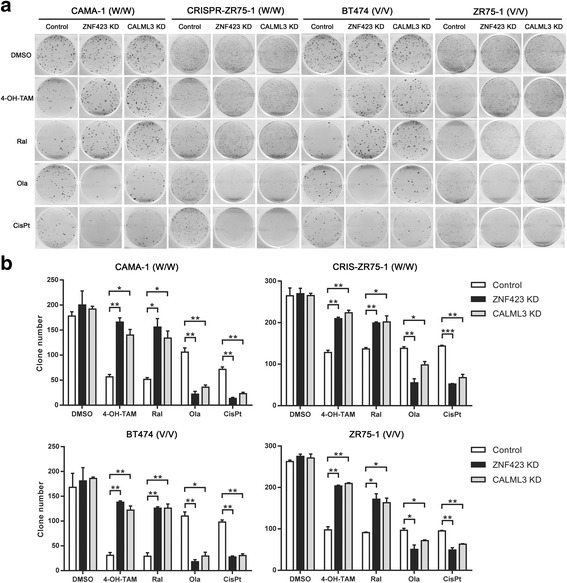
Colony formation in breast cancer cells with drug treatments after knocking down ZNF423 or CALML3. **a** Representative clones for breast cancer cells treated with drugs for 2–4 weeks. **b** Clone number counted in each well, shown as three independent experiments represented by mean ± SEM. Comparisons with the control for each treatment were performed by two-tailed Student’s *t* test. *p < 0.05, **p < 0.01. *DMSO* dimethylsulfoxide, *4-OH-TAM* 4-hydroxytamoxifene, *Ral* raloxifene, *Ola* olaparib, *CisPt* cisplatin

We have shown that BRCA1 is transcriptionally regulated by ZNF423. The SNP that resulted in the upregulation of ZNF423 and BRCA1 in the presence of tamoxifen was a protective SNP in our previous GWAS [8]. It is also known that patients deficient in BRCA1 display increased sensitivity to PARP inhibitors [26–28] or DNA-damaging agents such as platinum compounds [29, 30]. Therefore, we wanted to test whether the expression levels of ZNF423 and/or CALML3 might affect drug response by regulating the level of BRCA1. Knocking down ZNF423 or CALML3 altered the breast cancer cell response to several drugs including 4-OH-TAM, raloxifene, olaparib (PARP inhibitor) and cisplatin (platinum). Specifically, knocking down ZNF423 or CALML3 both caused cells to be more resistant to SERMs (Additional file 2: Figure S6A, B), whereas knocking down either of these two caused the cells to be more sensitive to olaparib (Additional file 2: Figure S6C) and cisplatin treatment (Additional file 2: Figure S6D). Similar results were also observed after knocking down ZNF423 and CALML3 in a panel of LCLs containing WT or variant *ZNF423* SNP genotypes (Additional file 2: Figure S7A–D). The cytotoxicity results were further confirmed by colony-forming assays. Compared to the controls, knocking down ZNF423 or CALML3 resulted in increased colony formation in the presence of E2 plus 4OH-TAM or raloxifene (Fig. 4a rows 2–3, 4b), whereas less colony formation was observed in the presence of olaparib or cisplatin (Fig. 4a rows 4–5, b). Moreover, overexpression of BRCA1 could reverse the effect on drug response induced by the downregulation of ZNF423 or CALML3 (Additional file 2: Figure S8), suggesting that the regulation of ZNF423 and CALML3 on drug response was mediated through their transcriptional regulation of BRCA1.

### The *ZNF423* rs9940645 SNP affects SERM and PARP inhibitor treatments

**Fig. 5:**
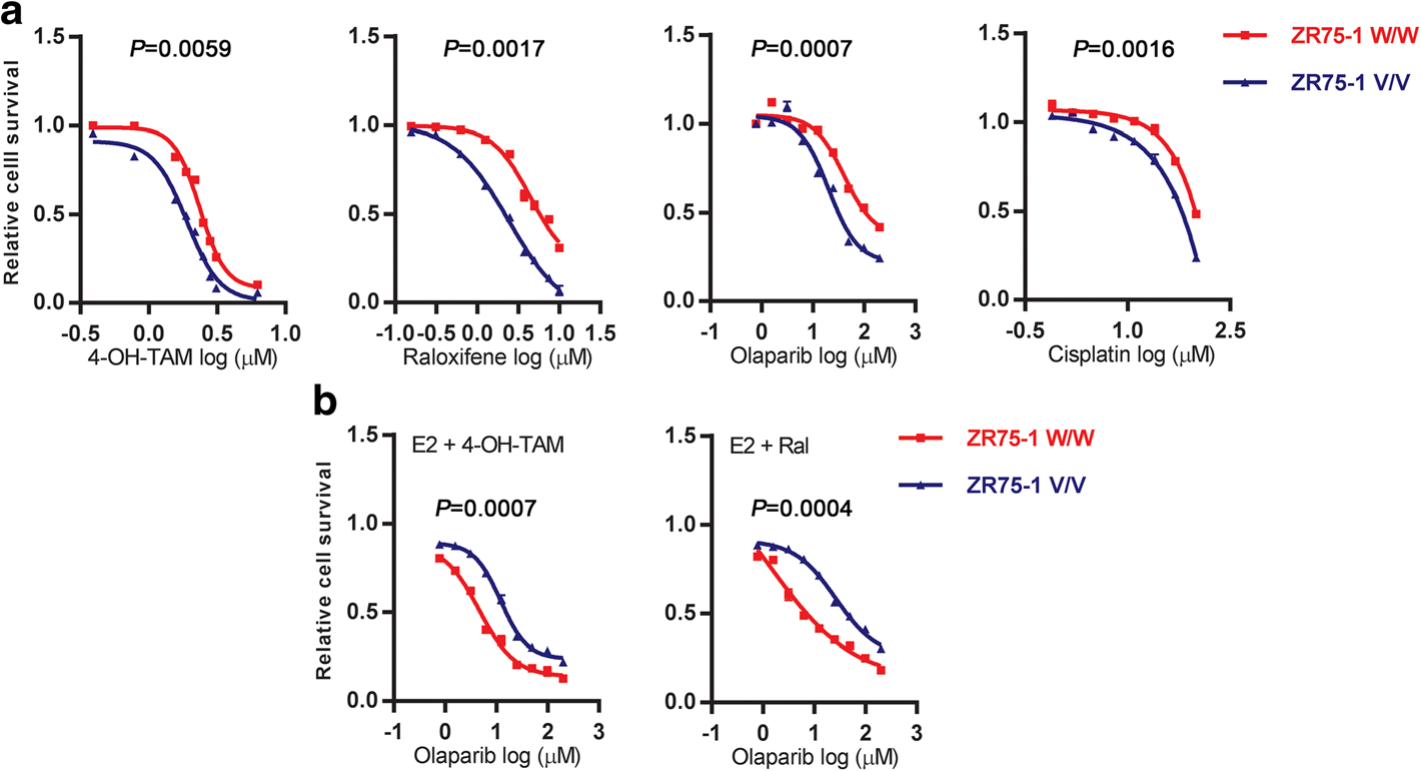
Cytotoxicity assays for drug treatments performed with CRISPR-ZR75-1 cells (WT) and ZR75-1 cells (variant). **a** Relative survival of CRISPR-ZR75-1 and ZR75-1 cells determined by MTS after treatment with 4-OH-TAM, raloxifene, olaparib or cisplatin for 3 days. **b** In the presence of 0.01 nM E2, cells were treated with 4-OH-TAM (1 μM) or raloxifene (3.16 μM) together with various concentrations of olaparib. Each data point of the variant cells was normalized as a percentage of their respective data point of the WT cells. Area under the curve was measured and comparisons were performed by unpaired *t* test. *p* values are indicated. *4-OH-TAM* 4-hydroxytamoxifene, *E2* estradiol, *Ral* raloxifene

Because the *ZNF423* rs9940645 SNP regulated the expression of ZNF423 and BRCA1, we next set out to test whether there was a difference in drug response between the WT and variant genotypes. In the ZR75-1 (V/V) and CRISPR-ZR75-1 (W/W) cell lines, there were differential responses to 4-OH-TAM, raloxifene, olaparib and cisplatin in the presence of E2 (Fig. 5a). As expected, cells containing the variant rs9940645 SNP genotype had a better response to 4-OH-TAM and raloxifene due to higher expression of ZNF423 and BRCA1. This result was consistent with the clinical observation in which patients carrying the variant SNP might benefit from treatment with these drugs. On the other hand, the variant SNP genotype also rendered cells more sensitive to olaparib and cisplatin, which might be due to the decreased expression of ZNF423 and BRCA1 in the presence of estrogen (Fig. 5a). We were able to reverse the drug response by combining SERMs with PARP inhibitor treatment, making the WT more sensitive to the PARP inhibitors (Fig. 5b). We also observed similar drug response phenotypes using LCL cells carrying either WT or variant rs9940645 genotypes (Additional file 2: Figure S7E).

**Fig. 6:**
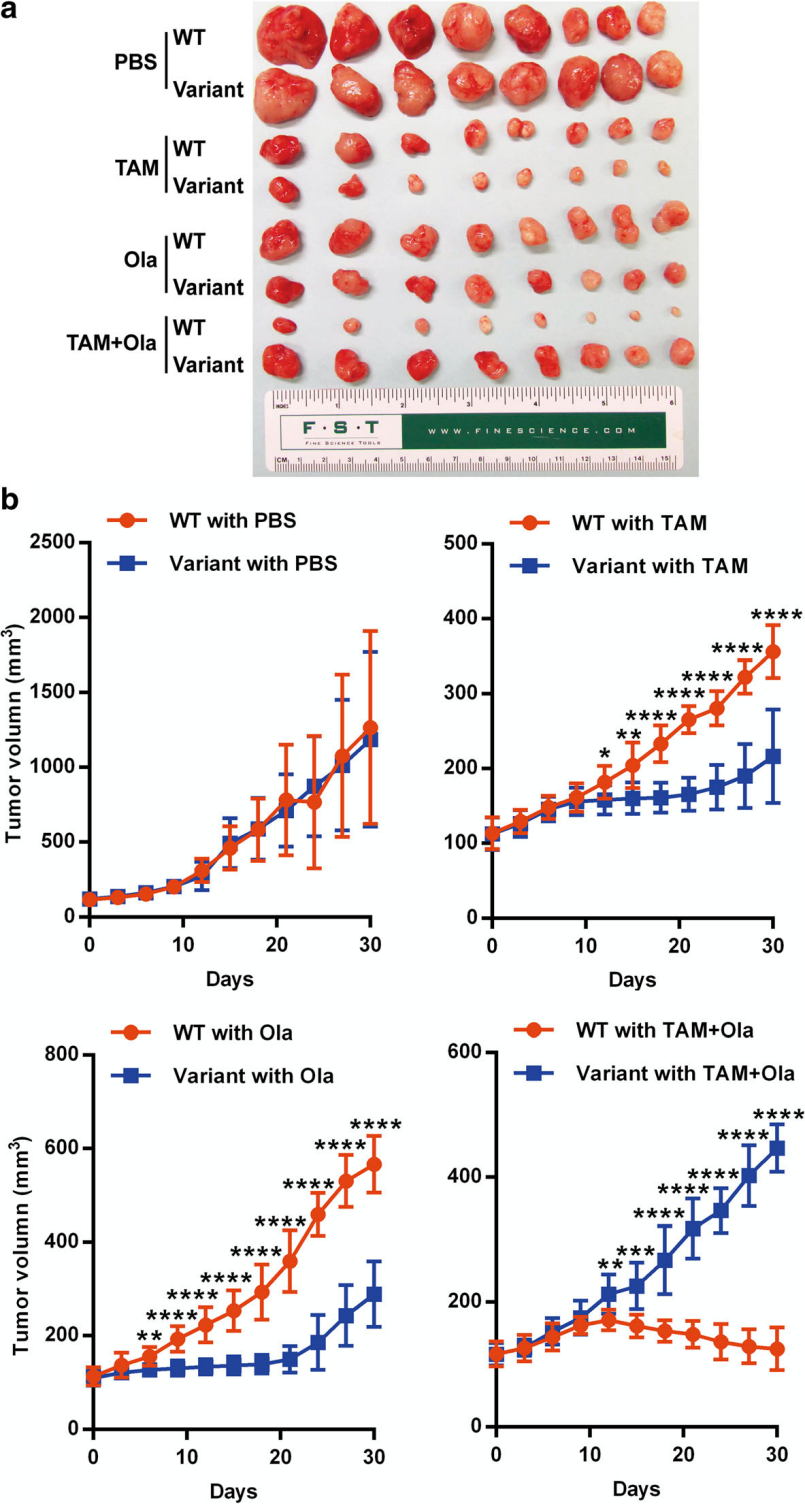
In-vivo study of drug response using cells carrying *ZNF423* WT (CRISPR-ZR75-1) or variant genotypes. **a** Tumors removed from the mice in each treatment group (eight mice) after 30 days of drug treatment. **b** Tumor volume measured every 3 days during treatment. Volume shown as mean ± SEM for eight mice, comparisons between WT and variant performed with two-tailed Student’s *t* test. *p < 0.05, **p < 0.01, ***p < 0.001, ****p < 0.0001. *Ola* olaparib, *PBS* phosphate-buffered saline, *Tam* tamoxifen, *WT* wild type

To further confirm the SNP effect on treatment response in vivo, we established a xenograft mouse model by injecting breast cancer cells ZR75-1 (V/V) and CRISPR-ZR75-1 (W/W) into female athymic nu/nu mice that had been water-fed with low-dose estrogen to stimulate tumor growth. The mice were randomized into four groups after the tumors reached 100 mm^3^, and were treated with tamoxifen, olaparib alone, olaparib in combination with tamoxifen or vehicle control PBS. Figure 6a shows the results on tumor growth after 30 days of treatment. There was no significant difference in tumor growth between the WT and variant genotypes with PBS treatment. Either tamoxifen or olaparib alone significantly inhibited the growth of tumors carrying the variant SNP genotype compared with the WT sequence. However, combining tamoxifen and olaparib dramatically inhibited the growth of tumors carrying the WT genotype (Fig. 6b). These results confirmed our observations in cell culture.

## Discussion

In the present study, we demonstrated that the *ZNF423* rs9940645 SNP, a SNP that was not in an ERE but rather approximately 200 bp away from the closest EREs, regulated ZNF423 and “downstream” BRCA1 expression in an estrogen and SERM-dependent fashion through CALML3, a potential new transcription factor (Figs. 2 and 3). The phenomenon that SNPs at a distance from EREs can modulate the binding of ERα to EREs has been observed previously [8, 31]. For example, SNPs in the *TCL1A* gene at a distance from EREs can regulate ERα binding and the expression of TCL1A [31], which are observations similar to those we have made for the *ZNF423* SNP. Our findings raise the possibility of a potentially common mechanism by which functional genetic variants close to EREs might influence nearby gene expression through a sensor protein that can cooperate with ERα to achieve SNP and hormone or drug-dependent transcriptional regulation. It is possible that other proteins might be also involved in the protein complex to coordinate ER activation. Our study also suggested that CALML3, working together with ERα, binds to the genomic region of the *ZNF423* promoter that contains the rs9940645 SNP involved in ZNF423 transcription regulation (Figs. 2 and 3). This regulation of CALML3 seems to be very specific for *ZNF423* because classical ERα-targeted gene expression was not affected in cells with knocked down of CALML3 (Additional file 2: Figure S5C). Future genome-wide ChIP assays with CALML3 antibody may better reveal its role as a potential transcription factor. We used both LCLs from healthy subjects with known germline SNP genotypes as well as breast cancer cells with heterogeneous genetic backgrounds to provide insight into the effect of the *ZNF423* SNP on gene transcriptional regulation. Furthermore, the effects of the SNP on ZNF423 and BRCA1 expression, as well as drug responses, were confirmed in cells with an isogenic genetic background, an observation which further emphasizes the importance of the functional implications of the *ZNF423* SNP and mechanisms by which the SNP influences gene transcription regulation. Based on our finding, CALML3 specifically bound to the region containing the SNP; however, little binding of ERα to this region was observed (Additional file 2: Figure S4B), further suggesting CALML3 as a sensor for the *ZNF423* SNP genotypes. Furthermore, CALML3 coordinates with ERα, regulating ZNF423 transcription, since knocking down of ERα which binds to the nearest EREs from the SNP reduced the CALML3–DNA binding.

Not only can the *ZNF423* SNP influence ZNF423 and BRCA1 expression through CALML3, but it can also affect cellular responses to anti-neoplastic drugs including 4-OH-TAM, raloxifene, olaparib and cisplatin (Fig. 4; Additional file 2: Figure S6). *BRCA1* appears to be one of many genes that might be regulated by ZNF423 [8, 32]. In our study, we found that the effects of CALML3 and ZNF423 on drug responses were probably mainly mediated through the regulation of BRCA1. As reported previously, tumors with deficient BRCA1 display defects in DNA homologous recombination repair and have increased sensitivity to DNA-damaging agents such as cisplatin [29, 30] and PARP inhibitors [26–28]. Our observations on drug responses after the downregulation of ZNF423 and CALML3 were consistent with effects associated with decreased BRCA1, implying that the effect of ZNF423 and CALMLs on drug responses might be mediated through BRCA1. Our rescue experiments also supported this hypothesis (Fig. 4; Additional file 2: Figure S8). The results of these experiments also suggest that common SNPs, in this case the *ZNF423* rs9940645 SNP, with a minor allele frequency (MAF) of 39% in Caucasian subjects in our GWAS [8], could result in biological effects similar to those of rare BRCA1 functional mutations or high CpG methylation of the BRCA1 promoter.

Our study has also raised the interesting possibility of using drugs to regulate endogenous gene expression by taking advantage of an individual’s genetic background. In this case, we have shown that SERMs can regulate BRCA1 expression in breast cancer cells based on the *ZNF423*SNP genotypes. Taking advantage of that observation, we successfully manipulated the response to a PARP inhibitor in cells carrying different *ZNF423* SNP genotypes. Specifically, cells with the *ZNF423* variant SNP genotype were sensitive to PARP inhibitor treatment alone whereas cells with the WT SNP genotype were more sensitive to the PARP inhibitor in combination with SERMs (Fig. 5). These cell line-based results were further confirmed by studies of our in-vivo xenograft model (Fig. 6). Of importance, both E2 and drug concentrations used in these studies were within physiological and therapeutic ranges [33–35].

Our observations suggest that we may be able to use PARP inhibitors, either alone or in combination with SERMs, to treat selected patients based on their *ZNF423* genotypes. Many breast cancer patients likely do not have functional *BRCA1* mutations or abnormal *BRCA1* CpG methylation, but since the MAF of the *ZNF423* SNP is relatively high, the genotype for this SNP could be a useful factor in the selection of different treatments and could have potentially broad clinical implications.

In the current study, we focused on the common proteins identified by MS in three DNA–protein complexes based on our EMSA study in order to have high confidence of identifying functional candidates in our EMSA bands (Fig. 1). We understand that we might miss other proteins which may be transcription regulators of ZNF423. Among the proteins that were common to all three EMSA bands, CALML3 is the only SNP binding protein which can be experimentally validated using specific antibodies. In the future, more work is required to thoroughly examine the transcription regulation of ZNF423. For the mechanistic basis of the SNP and drug-dependent regulation of ZNF423 expression, we hypothesize that different receptor conformations upon ligand binding might have different affinity to bind the genomic regions containing different SNP genotypes. At the same time, this process might attract different transcription coregulators to the region, and together the protein complexes can regulate nearby gene transcription in a SNP and ligand-dependent fashion. Obviously, this hypothesis needs to be further tested in the future.

In the available public databases, we did not find predicted classical DNA binding domains in *CALML3* gene. It is possible that CALML3 may bind to the DNA through an unconventional DNA binding motif, or indirectly through tethering to another DNA-bound target protein which may not be identified by our EMSA/MS. Further deletion experiments of CALML3 are required to define specific DNA binding regions. Although CALML3 binding to the *ZNF423* SNP is calcium independent (Additional file 2: Figure S5B), the interaction of CALML3 with ERα is calcium dependent (Additional file 2: Figure S5A). The function of CALML3 is similar to another calcium family member calmodulin (CaM), which has high similarity with CALML3, as calcium is also essential for ER interaction with CaM and activation [36–38]. This also explains why ER was not identified in our EMSA/MS (Additional file 1). ERα interaction with CALML3 requires calcium but the binding reaction of our EMSA was performed under calcium-free conditions.

Finally, in order to fully understand the role of ZNF423 in either breast cancer risk or response to anti-neoplastic drug therapy, additional experiments will need to be performed to identify additional ZNF423-regulated genes or pathways which could be regulated in a similar SNP and drug-dependent fashion. Regulation might have additional important therapeutic or diagnostic implications.

## Conclusions

We have identified CALML3 as a sensor of the *ZNF423* rs9940645 SNP and as a coregulator of ERα in response to estrogen and SERM treatment. The *ZNF423* SNP affected drug response by regulating the level of expression of ZNF423 and BRCA1, and as a result the SNP could be a potential biomarker for tailoring drug treatment.

## Additional files

Additional file 1: Presents the MS results. (PDF 8620 kb)

Additional file 2: Figures S1-S8. showing supplementary results. (PDF 10332 kb)

## Abbreviations

4-OH-TAM: 4-Hydroxytamoxifene
ERE: Estrogen response element
GWAS: Genome-wide association study
LCL: Lymphoblastoid cell line
NSABP: National Surgical Adjuvant Breast and Bowel Project
PARP: Poly(ADP-ribose) polymerase
Ral: Raloxifene
SERM: Selective estrogen receptor modulator
SNP: Single nucleotide polymorphism
WT: Wild type
