# Supplementary material for: Calmodulin-like protein 3 is an estrogen receptor alpha coregulator for gene expression and drug response in a SNP, estrogen, and SERM-dependent fashion
Source: Breast Cancer Res. 2017 Aug 18;19:95. doi: 10.1186/s13058-017-0890-x (PMC5562991; doi:10.1186/s13058-017-0890-x)

Mass spectrometry for Band 1

The band indicating binding between nuclear extract of *ZNF423* WT LCLs treated with E2 and WT DNA probe in Figure 1B

Protein matches	☐	scanf	Rank_Sp	charge	Ions	dCn	dCn2	XCorr	Reference	Redu	Peptide
**9 9** 3.253 0.00 CCT8_IPI:IPI00302925.4	☐	1615	1/1	2	23/28	0.4274	0.4274	4.2806	CCT8_IPI:IPI00302925.4	2	K.AIADTGA
**9 9** 3.087 0.00 HSP90B1_IPI:IPI00027230.3	☐	0751	1/110	2	10/14	0.1064	0.1064	2.0677	CCT8_IPI:IPI00302925.4	2	K.DWDDDQ
**8 8** 3.806 0.00 HSP90AA1_IPI:IPI00382470.3	☐	0569	1/1	2	14/18	0.2720	0.2720	2.3271	CCT8_IPI:IPI00302925.4	1	K.ETEGDVT
**6 6** 4.365 0.00 PDIA6_IPI:IPI00299571.5	☐	1027	1/1	2	17/20	0.2810	0.2810	3.8979	CCT8_IPI:IPI00302925.4	2	K.GEENLM*
**6 6** 3.934 0.00 HSPA5_IPI:IPI00003362.2	☐	2199	1/3	2	16/20	0.1089	0.1089	2.4781	CCT8_IPI:IPI00302925.4	2	K.LATNAAV
**6 6** 3.312 0.00 VCP_IPI:IPI00022774.3	☐	2820	1/1	2	19/22	0.3041	0.3041	3.4342	CCT8_IPI:IPI00302925.4	2	K.LFVTNDA
**5 5** 3.243 0.00 EIF4A1_IPI:IPI00025491.1	☐	2514	1/1	2	20/26	0.3881	0.3881	4.1630	CCT8_IPI:IPI00302925.4	2	K.NVGLDIE
**4 4** 3.592 0.00 DYNC1I2_IPI:IPI00216348.1	☐	1450	1/1	2	16/18	0.3456	0.3456	3.2902	CCT8_IPI:IPI00302925.4	1	R.AVDDGVI
**4 4** 3.590 0.00 ANXA6_IPI:IPI00002459.4	☐	2570	1/7	2	15/18	0.2050	0.2050	3.3397	CCT8_IPI:IPI00302925.4	2	R.DIDEVSSI
**3 3** 4.605 0.00 MYH9_IPI:IPI00019502.3	☐	0330	1/5	2	10/14	0.0857	0.0857	1.8928	HSP90B1_IPI:IPI00027230.3	1	K.AQAYQTG
**3 3** 4.241 0.00 ACTN4_IPI:IPI00013808.1	☐	2326	1/1	2	15/24	0.3505	0.3505	3.9053	HSP90B1_IPI:IPI00027230.3		K.EEASDYL
**3 3** 3.617 0.00 MCM3_IPI:IPI00013214.2	☐	0989	1/11	2	11/16	0.0790	0.0790	1.9420	HSP90B1_IPI:IPI00027230.3	1	K.ESTAEKD
**3 3** 3.585 0.00 HSP90AA2_IPI:IPI00031523.4	☐	2234	1/1	2	22/26	0.4437	0.4437	4.7103	HSP90B1_IPI:IPI00027230.3		K.GVVDSDI
**3 3** 3.353 0.00 ANXA2P2_IPI:IPI00334627.3	☐	1551	1/1	2	15/16	0.1975	0.1975	2.7947	HSP90B1_IPI:IPI00027230.3		K.SGTSEFLI
**3 3** 3.194 0.00 ACTA2_IPI:IPI00008603.1	☐	2703	1/9	2	14/20	0.1154	0.1154	2.7321	HSP90B1_IPI:IPI00027230.3		K.SILFVPTS
**3 3** 2.973 0.00 HNRNPK_IPI:IPI00216049.1	☐	1952	1/1	2	19/22	0.3501	0.3501	4.0420	HSP90B1_IPI:IPI00027230.3	6	R.ELISNASD
**3 3** 2.846 0.00 CALML3_IPI:IPI00216984.5	☐	2100	1/7	2	13/18	0.1572	0.1572	2.9496	HSP90B1_IPI:IPI00027230.3		R.LSLNIDPD
**3 3** 2.300 0.00 HTATSF1_IPI:IPI00013788.1	☐	2009	1/2	2	14/16	0.2218	0.2218	2.8121	HSP90B1_IPI:IPI00027230.3		R.SGYLLPD
**2 2** 4.025 0.00 COPB2_IPI:IPI00220219.6	☐	1466	1/2	2	16/20	0.3187	0.3187	3.7371	HSP90AA1_IPI:IPI00382470.3	2	K.DQVANSA
**2 2** 3.806 0.00 ACTN1_IPI:IPI00013508.5	☐	1206	1/1	2	15/18	0.3020	0.3020	3.3312	HSP90AA1_IPI:IPI00382470.3	5	K.EDQTEYL
**2 2** 3.597 0.00 CDC37_IPI:IPI00013122.1	☐	1721	1/1	2	18/22	0.2541	0.2541	3.2268	HSP90AA1_IPI:IPI00382470.3	6	K.EGLELPEI
**2 2** 3.526 0.00 HSP90AB1_IPI:IPI00411633.4	☐	2295	1/1	3	37/132	0.4565	0.4565	6.9092	HSP90AA1_IPI:IPI00382470.3	2	K.LGLGIDEI
**2 2** 3.477 0.00 LRPPRC_IPI:IPI00783271.1	☐	0550	1/1	2	14/20	0.2440	0.2440	2.8321	HSP90AA1_IPI:IPI00382470.3	2	R.DNSTM*G
**2 2** 3.402 0.00 ACTBL2_IPI:IPI00003269.1	☐	2458	1/2	2	18/26	0.3153	0.3153	3.7699	HSP90AA1_IPI:IPI00382470.3	7	R.GVVDSED
**2 2** 3.366 0.00 HSP90AB1_IPI:IPI00414676.6	☐	2171	1/1	2	23/28	0.4230	0.4230	4.9528	HSP90AA1_IPI:IPI00382470.3	2	R.NPDDITNI
**2 2** 3.191 0.00 ACTB_IPI:IPI00021439.1	☐	0282	1/4	2	12/16	0.1177	0.1177	1.6915	HSP90AA1_IPI:IPI00382470.3	1	R.TDTGEPM
**2 2** 3.161 0.00 IVL_IPI:IPI00011692.2	☐	2345	1/1	2	19/22	0.2701	0.2701	3.6792	PDIA6_IPI:IPI00299571.5	1	K.DVIELTDI
**2 2** 2.948 0.00 SND1_IPI:IPI00140420.4	☐	1019	1/1	2	15/20	0.3280	0.3280	3.0856	PDIA6_IPI:IPI00299571.5	1	K.GESPVDY
**2 2** 2.918 0.00 XRCC6_IPI:IPI00644712.4	☐	2712	1/1	3	33/56	0.3480	0.3480	5.5163	PDIA6_IPI:IPI00299571.5	1	K.LAAVDAT
**1 1** 5.838 0.00 IPI:IPI00550731.2_IPI:IPI00550731.2	☐	3066	1/1	3	30/96	0.2790	0.2790	5.2737	PDIA6_IPI:IPI00299571.5	1	R.DGELPVE
**1 1** 5.320 0.00 YWHAZ_IPI:IPI00021263.3	☐	2373	1/1	2	25/32	0.4917	0.4917	4.4088	PDIA6_IPI:IPI00299571.5	1	R.GSTAPVG
**1 1** 5.315 0.00 ANXA6_IPI:IPI00221226.7	☐	3258	1/1	2	21/26	0.3114	0.3114	4.2278	PDIA6_IPI:IPI00299571.5	1	R.TGEAIVD.
**1 1** 5.285 0.00 HNRNPH1_IPI:IPI00013881.6	☐	2530	1/1	2	18/22	0.3176	0.3176	4.5907	HSPA5_IPI:IPI00003362.2		K.ELEEIVQI
**1 1** 5.220 0.00 ILF2_IPI:IPI00005198.2	☐	0903	1/12	2	12/16	0.1049	0.1049	1.9677	HSPA5_IPI:IPI00003362.2		K.ITITNDQN
**1 1** 5.024 0.00 LDHA_IPI:IPI00217966.8	☐	2067	1/1	2	22/28	0.3510	0.3510	4.4687	HSPA5_IPI:IPI00003362.2		K.NQLTSNP
**1 1** 4.941 0.00 BAT1_IPI:IPI00641829.5	☐	2447	1/1	2	20/26	0.4046	0.4046	3.8312	HSPA5_IPI:IPI00003362.2		K.SDIDEIVL
**1 1** 4.869 0.00 YWHAQ_IPI:IPI00018146.1	☐	2105	1/1	2	27/32	0.4636	0.4636	5.4106	HSPA5_IPI:IPI00003362.2		K.SQIFSTAS
**1 1** 4.706 0.00 ANXA1_IPI:IPI00218918.5	☐	1280	1/1	2	19/20	0.1929	0.1929	3.3366	HSPA5_IPI:IPI00003362.2	15	R.VEIIANDC
**1 1** 4.527 0.00 ALDOC_IPI:IPI00418262.5	☐	2601	1/1	2	13/16	0.1267	0.1267	2.1621	VCP_IPI:IPI00022774.3		K.DVDLEFL
**1 1** 4.388 0.00 XRCC5_IPI:IPI00220834.8	☐	2094	1/1	2	16/18	0.2698	0.2698	3.1480	VCP_IPI:IPI00022774.3		K.GDDLSTA
**1 1** 4.332 0.00 ENO1_IPI:IPI00465248.5	☐	1116	1/3	2	13/18	0.1420	0.1420	2.3714	VCP_IPI:IPI00022774.3		K.LAGESES'
**1 1** 4.300 0.00 TPI1_IPI:IPI00383071.1	☐	1992	1/1	2	18/22	0.2436	0.2436	3.1858	VCP_IPI:IPI00022774.3		R.EVDIGIPD
**1 1** 4.276 0.00 TPM2_IPI:IPI00013991.1	☐	0873	1/1	2	20/24	0.2484	0.2484	3.3129	VCP_IPI:IPI00022774.3		R.GGNIGDG
**1 1** 4.235 0.00 HNRNPF_IPI:IPI00003881.5	☐	2549	1/1	2	26/38	0.4573	0.4573	5.6904	VCP_IPI:IPI00022774.3		R.LIVDEAIN
**1 1** 4.135 0.00 TUBB2C_IPI:IPI00007752.1	☐	1925	1/1	2	16/20	0.3201	0.3201	3.1032	EIF4A1_IPI:IPI00025491.1	2	K.ATQALVI
**1 1** 4.001 0.00 RPLP2_IPI:IPI00008529.1	☐	2227	1/1	2	15/16	0.1569	0.1569	2.5072	EIF4A1_IPI:IPI00025491.1	2	K.EELTLEG
**1 1** 3.676 0.00 RPS17_IPI:IPI00221093.7	☐	1744	1/1	2	23/26	0.4747	0.4747	5.0549	EIF4A1_IPI:IPI00025491.1	9	K.GYDVIAQ
**1 1** 3.664 0.00 RPLP0_IPI:IPI00008530.1	☐	1148	1/51	2	11/14	0.0586	0.0586	2.4043	EIF4A1_IPI:IPI00025491.1	8	R.ELAQIQY
**1 1** 3.485 0.00 HNRNPA2B1_IPI:IPI00386854.6	☐	2796	1/1	2	17/18	0.2642	0.2642	3.1431	EIF4A1_IPI:IPI00025491.1	8	R.VLITTDLI
**1 1** 3.337 0.00 HSPA7_IPI:IPI00011134.2	☐	1470	1/1	2	18/26	0.4064	0.4064	3.5396	DYNC1I2_IPI:IPI00216348.1	17	K.EAVAPVC
**1 1** 3.123 0.00 TPM1_IPI:IPI00014581.1	☐	0901	1/1	2	25/40	0.5350	0.5350	5.1412	DYNC1I2_IPI:IPI00216348.1	16	K.SVSTPSEA
**1 1** 3.091 0.00 IGLC1_IPI:IPI00154742.6	☐	0438	1/1	2	17/18	0.2392	0.2392	3.8373	DYNC1I2_IPI:IPI00216348.1	6	R.ADAEEEA
**1 1** 3.089 0.00 LMNA_IPI:IPI00021405.3	☐	1067	1/17	2	10/12	0.0617	0.0617	1.8488	DYNC1I2_IPI:IPI00216348.1	12	R.EIVTYTK.
**1 1** 3.072 0.00 TUBA1C_IPI:IPI00166768.3	☐	1221	1/2	2	14/20	0.1453	0.1453	2.8479	ANXA6_IPI:IPI00002459.4	1	K.DAISGIGT
**1 1** 3.047 0.00 EIF5_IPI:IPI00022648.2	☐	2872	1/1	2	17/20	0.2873	0.2873	3.6575	ANXA6_IPI:IPI00002459.4	2	R.DAFVAIV
**1 1** 2.964 0.00 TPM1_IPI:IPI00000230.6	☐	3016	1/1	3	37/96	0.4054	0.4054	5.9086	ANXA6_IPI:IPI00002459.4	1	R.EEDDVVS
**1 1** 2.814 0.00 NPM1_IPI:IPI00220740.1	☐	2677	1/2	2	14/16	0.2300	0.2300	1.9465	ANXA6_IPI:IPI00002459.4	2	R.SEIDLLNI

Mass spectrometry for Band 2

The band indicating binding between nuclear extract of *ZNF423* variant LCLs treated with E2 and variant DNA probe in Figure 1B

Protein matches	☐	scanf	Rank_Sp	charge	Ions	dCn	dCn2	XCorr	Reference	Redu	Peptide
**7 7** 4.550 0.00 MYH9_IPI:IPI00019502.3	☐	3208	1/1	2	23/30	0.4614	0.4614	5.0631	MYH9_IPI:IPI00019502.3	2	K.ANLQIDQ
**5 5** 2.800 0.00 NCL_IPI:IPI00444262.3	☐	2181	1/1	2	15/16	0.2889	0.2889	2.8423	MYH9_IPI:IPI00019502.3	2	K.ASITALE
**4 4** 4.231 0.00 PDIA6_IPI:IPI00299571.5	☐	2748	1/1	2	20/24	0.3669	0.3669	4.2381	MYH9_IPI:IPI00019502.3	2	K.IAQLEEQ
**4 4** 3.826 0.00 HSPA5_IPI:IPI00003362.2	☐	3450	1/1	2	27/34	0.5040	0.5040	6.3822	MYH9_IPI:IPI00019502.3		K.LQVELD
**4 4** 3.650 0.00 CALML3_IPI:IPI00216984.5	☐	1286	1/1	2	21/26	0.5305	0.5305	4.9696	MYH9_IPI:IPI00019502.3	2	R.ELEDATE
**4 4** 3.004 0.00 ACTA2_IPI:IPI00008603.1	☐	1904	1/1	2	22/26	0.5142	0.5142	4.6837	MYH9_IPI:IPI00019502.3	2	R.ELEDATE
**4 4** 2.935 0.00 ATP5A1_IPI:IPI00440493.2	☐	1379	1/1	2	18/20	0.2707	0.2707	3.6733	MYH9_IPI:IPI00019502.3	2	R.QLEEAEE
**3 3** 3.758 0.00 LMNA_IPI:IPI00021405.3	☐	3002	1/3	2	12/14	0.0780	0.0780	2.4773	NCL_IPI:IPI00444262.3	2	K.ALELTGL
**3 3** 3.244 0.00 TUBA1C_IPI:IPI00166768.3	☐	2727	1/2	2	15/18	0.2198	0.2198	2.8566	NCL_IPI:IPI00444262.3	3	K.EVFEDAA
**3 3** 2.927 0.00 HSPB1_IPI:IPI00025512.2	☐	2538	1/1	2	15/16	0.2600	0.2600	3.6681	NCL_IPI:IPI00444262.3	2	K.NDLAVVI
**3 3** 2.853 0.00 CKAP4_IPI:IPI00141318.2	☐	2968	1/1	2	14/16	0.1986	0.1986	2.7748	NCL_IPI:IPI00444262.3	2	K.TGISDVF
**3 3** 2.777 0.00 NCL_IPI:IPI00183526.6	☐	0740	1/103	2	9/12	0.0638	0.0638	2.2209	NCL_IPI:IPI00444262.3	3	K.VTQDELK
**3 3** 2.725 0.00 GSN_IPI:IPI00026314.1	☐	3410	1/1	2	23/28	0.4767	0.4767	4.9983	PDIA6_IPI:IPI00299571.5	1	K.LAAVDA
**2 2** 5.798 0.00 MYH14_IPI:IPI00029818.5	☐	3399	1/1	2	20/26	0.2109	0.2109	3.5511	PDIA6_IPI:IPI00299571.5	1	K.NLEPEW
**2 2** 3.916 0.00 IL1F9_IPI:IPI00021343.1	☐	3076	1/1	2	22/32	0.4552	0.4552	4.2233	PDIA6_IPI:IPI00299571.5	1	R.GSTAPVG
**2 2** 3.670 0.00 ANXA3_IPI:IPI00024095.3	☐	3846	1/1	2	22/26	0.2781	0.2781	4.1517	PDIA6_IPI:IPI00299571.5	1	R.TGEAIVD
**2 2** 3.603 0.00 TUBB_IPI:IPI00011654.2	☐	3230	1/1	2	19/22	0.3155	0.3155	4.4695	HSPA5_IPI:IPI00003362.2		K.ELEEIVQ
**2 2** 3.543 0.00 FLNA_IPI:IPI00302592.2	☐	1188	1/3	2	12/16	0.1684	0.1684	2.1747	HSPA5_IPI:IPI00003362.2		K.ITITNDQ
**2 2** 3.391 0.00 ATP5B_IPI:IPI00303476.1	☐	2698	1/1	2	27/32	0.4469	0.4469	5.9218	HSPA5_IPI:IPI00003362.2		K.SQIFSTAS
**2 2** 3.376 0.00 HSP90AA2_IPI:IPI00031523.4	☐	3406	1/1	2	19/30	0.1957	0.1957	2.7386	HSPA5_IPI:IPI00003362.2	8	R.IINEPTAA
**2 2** 3.337 0.00 ANXA2P2_IPI:IPI00334627.3	☐	0640	1/5	2	14/16	0.1443	0.1443	3.2410	CALML3_IPI:IPI00216984.5		K.DTDNEE
**2 2** 2.877 0.00 NPM1_IPI:IPI00220740.1	☐	1828	1/2	2	15/20	0.2486	0.2486	3.1524	CALML3_IPI:IPI00216984.5		K.LSDEEVD
**2 2** 2.680 0.00 SH3BGRL3_IPI:IPI00010402.2	☐	2712	1/1	2	21/32	0.3992	0.3992	5.0832	CALML3_IPI:IPI00216984.5		R.AADTDG
**2 2** 2.575 0.00 HSP90B1_IPI:IPI00027230.3	☐	2425	1/1	2	18/22	0.3223	0.3223	3.1234	CALML3_IPI:IPI00216984.5		R.SLGQNPT
**1 1** 5.717 0.00 PKM2_IPI:IPI00220644.8	☐	1914	1/1	2	16/18	0.3412	0.3412	2.9343	ACTA2_IPI:IPI00008603.1	31	K.AGFAGD
**1 1** 5.066 0.00 ACTN4_IPI:IPI00013808.1	☐	0858	1/1	2	18/20	0.0688	0.0688	3.4745	ACTA2_IPI:IPI00008603.1	23	K.DSYVGD
**1 1** 5.054 0.00 YWHAZ_IPI:IPI00021263.3	☐	2025	1/1	2	15/20	0.2568	0.2568	3.2854	ACTA2_IPI:IPI00008603.1	18	K.EITALAP
**1 1** 5.034 0.00 HSP90AA1_IPI:IPI00382470.3	☐	2523	1/3	2	11/20	0.1066	0.1066	2.3233	ACTA2_IPI:IPI00008603.1	18	K.EITALAP
**1 1** 4.947 0.00 ANXA2_IPI:IPI00418169.3	☐	3010	1/24	2	12/18	0.2874	0.2874	2.6749	ATP5A1_IPI:IPI00440493.2	3	K.AVDSLV
**1 1** 4.909 0.00 EIF6_IPI:IPI00010105.1	☐	2846	1/1	2	22/28	0.3094	0.3094	3.6567	ATP5A1_IPI:IPI00440493.2	4	R.ILGADTS
**1 1** 4.908 0.00 HSPD1_IPI:IPI00784154.1	☐	3546	1/7	2	15/30	0.0714	0.0714	3.1978	ATP5A1_IPI:IPI00440493.2	3	R.TGAIVDV
**1 1** 4.798 0.00 ACTBL2_IPI:IPI00003269.1	☐	0407	1/1	2	13/14	0.1754	0.1754	2.2116	ATP5A1_IPI:IPI00440493.2	1	R.VGSAAQ
**1 1** 4.708 0.00 RPLP2_IPI:IPI00008529.1	☐	1258	1/1	2	17/18	0.2789	0.2789	3.6558	LMNA_IPI:IPI00021405.3	5	R.ITESEEV
**1 1** 4.402 0.00 TGM1_IPI:IPI00305622.4	☐	1546	1/1	2	20/26	0.2489	0.2489	4.1514	LMNA_IPI:IPI00021405.3	5	R.SGAQASS
**1 1** 4.376 0.00 SFN_IPI:IPI00013890.2	☐	2040	1/1	2	17/18	0.3166	0.3166	3.4677	LMNA_IPI:IPI00021405.3	5	R.SLETENA
**1 1** 4.201 0.00 BTF3_IPI:IPI00221035.4	☐	2441	1/1	2	17/18	0.3432	0.3432	3.2113	TUBA1C_IPI:IPI00166768.3	9	K.DVNAAIA
**1 1** 4.178 0.00 GSDMA_IPI:IPI00166200.4	☐	3595	1/1	2	13/16	0.1024	0.1024	2.2405	TUBA1C_IPI:IPI00166768.3	8	K.EIIDLVL
**1 1** 4.122 0.00 ERO1L_IPI:IPI00386755.2	☐	3602	1/1	2	20/28	0.3290	0.3290	4.2815	TUBA1C_IPI:IPI00166768.3	9	R.AVFVDL
**1 1** 4.115 0.00 GAPDH_IPI:IPI00219018.7	☐	3161	1/1	2	20/32	0.4179	0.4179	3.9422	HSPB1_IPI:IPI00025512.2	1	K.LATQSN
**1 1** 4.093 0.00 PHB_IPI:IPI00017334.1	☐	1342	1/2	2	14/18	0.2200	0.2200	2.3779	HSPB1_IPI:IPI00025512.2	1	R.AQLGGP
**1 1** 3.893 0.00 PSME3_IPI:IPI00030243.1	☐	2198	1/14	2	12/18	0.2322	0.2322	2.4606	HSPB1_IPI:IPI00025512.2	2	R.QLSSGVS
**1 1** 3.874 0.00 TPI1_IPI:IPI00383071.1	☐	3273	1/1	2	13/16	0.1172	0.1172	2.2550	CKAP4_IPI:IPI00141318.2	2	R.LALQALT
**1 1** 3.805 0.00 ACTN1_IPI:IPI00013508.5	☐	3248	1/1	2	15/20	0.2093	0.2093	2.4614	CKAP4_IPI:IPI00141318.2	2	R.QTESLES
**1 1** 3.677 0.00 VCL_IPI:IPI00291175.7	☐	2872	1/1	2	19/26	0.3098	0.3098	3.8415	CKAP4_IPI:IPI00141318.2	2	R.SVGELPS
**1 1** 3.647 0.00 ATP6V1A_IPI:IPI00007682.2	☐	1800	1/1	2	17/22	0.3722	0.3722	3.6486	NCL_IPI:IPI00183526.6	4	K.GLSEDTT
**1 1** 3.617 0.00 FLNB_IPI:IPI00289334.1	☐	1061	1/2	2	13/16	0.1854	0.1854	2.6978	NCL_IPI:IPI00183526.6	3	K.NSTWSG
**1 1** 3.577 0.00 HNRNPR_IPI:IPI00012074.3	☐	0437	1/25	2	11/14	0.2020	0.2020	1.9852	NCL_IPI:IPI00183526.6	4	K.QGTEIDG
**1 1** 3.546 0.00 LOC389842_IPI:IPI00399212.3	☐	0410	1/1	2	16/20	0.3138	0.3138	3.0227	GSN_IPI:IPI00026314.1	4	R.EGGQTA
**1 1** 3.509 0.00 IPI:IPI00550731.2_IPI:IPI00550731.2	☐	0425	1/9	2	13/20	0.1694	0.1694	2.7006	GSN_IPI:IPI00026314.1		R.GASQAG
**1 1** 3.506 0.00 RBBP4_IPI:IPI00328319.8	☐	1365	1/31	2	11/16	0.1827	0.1827	2.4530	GSN_IPI:IPI00026314.1	4	R.YIETDPA
**1 1** 3.397 0.00 HNRNPM_IPI:IPI00171903.2	☐	2791	1/1	2	27/34	0.5100	0.5100	5.8270	MYH14_IPI:IPI00029818.5	3	R.AQAELEN
**1 1** 3.310 0.00 ENO1_IPI:IPI00465248.5	☐	2980	1/1	2	25/32	0.4466	0.4466	5.7700	MYH14_IPI:IPI00029818.5	4	R.EAQAAL
**1 1** 3.302 0.00 ACTB_IPI:IPI00021439.1	☐	2984	1/1	2	16/22	0.2511	0.2511	4.1907	IL1F9_IPI:IPI00021343.1	1	K.SYNTAFE
**1 1** 3.279 0.00 TPM3_IPI:IPI00218319.3	☐	3130	1/1	2	19/22	0.1863	0.1863	3.6421	IL1F9_IPI:IPI00021343.1	1	R.DQPIILTS
**1 1** 3.267 0.00 HMGB3L1_IPI:IPI00006437.2	☐	3716	1/1	2	17/30	0.3010	0.3010	3.8315	ANXA3_IPI:IPI00024095.3	1	K.GAGTNE
**1 1** 3.257 0.00 EIF3K_IPI:IPI00033143.1	☐	2733	1/1	2	18/28	0.3791	0.3791	3.5081	ANXA3_IPI:IPI00024095.3	1	K.SLGDDIS
**1 1** 3.240 0.00 MYL6B_IPI:IPI00027255.1	☐	3375	1/1	2	21/28	0.3823	0.3823	4.1298	TUBB_IPI:IPI00011654.2	7	R.AILVDLE
**1 1** 3.187 0.00 FSCN1_IPI:IPI00163187.1	☐	3588	1/1	2	17/28	0.2490	0.2490	3.0755	TUBB_IPI:IPI00011654.2	4	R.ALTVPEL
**1 1** 3.172 0.00 EIF4A1_IPI:IPI00025491.1	☐	3542	1/1	3	34/92	0.3756	0.3756	4.5818	FLNA_IPI:IPI00302592.2	4	K.ASGPGLN
**1 1** 3.093 0.00 PGK1_IPI:IPI00169383.3	☐	1709	1/5	2	14/18	0.1009	0.1009	2.5041	FLNA_IPI:IPI00302592.2	4	K.GTVEPQL
**1 1** 3.047 0.00 TPM1_IPI:IPI00000230.6	☐	3428	1/1	2	14/18	0.2545	0.2545	2.8469	ATP5B_IPI:IPI00303476.1	1	K.VVDLLA
**1 1** 3.006 0.00 NCCRP1_IPI:IPI00247167.3	☐	3526	1/1	2	20/26	0.4248	0.4248	3.9360	ATP5B_IPI:IPI00303476.1		R.FTQAGSE
**1 1** 3.005 0.00 ANXA8L1_IPI:IPI00414499.3	☐	3238	1/1	2	19/22	0.2737	0.2737	3.7245	HSP90AA2_IPI:IPI00031523.4	6	K.ADLINNL
**1 1** 2.986 0.00 ATP5D_IPI:IPI00024920.1	☐	1806	1/15	2	13/16	0.0793	0.0793	3.0282	HSP90AA2_IPI:IPI00031523.4	5	K.YIDQEEL
**1 1** 2.969 0.00 HIST1H4J_IPI:IPI00453473.6	☐	2807	1/1	2	15/18	0.1912	0.1912	3.4635	ANXA2P2_IPI:IPI00334627.3	5	R.DALNIET
**1 1** 2.955 0.00 RPL22_IPI:IPI00219153.4	☐	1894	1/1	2	15/18	0.2493	0.2493	3.2112	ANXA2P2_IPI:IPI00334627.3	5	R.TNQELQE
**1 1** 2.886 0.00 HSPD1_IPI:IPI00076042.2	☐	1530	1/5	2	13/16	0.1867	0.1867	2.3502	NPM1_IPI:IPI00220740.1	2	K.GPSSVED
**1 1** 2.879 0.00 DLD_IPI:IPI00015911.1	☐	3031	1/1	2	16/24	0.2799	0.2799	3.4046	NPM1_IPI:IPI00220740.1	5	K.VDNDEN
**1 1** 2.852 0.00 KLK6_IPI:IPI00023845.1	☐	0382	1/3	2	12/14	0.1129	0.1129	2.4002	SH3BGRL3_IPI:IPI00010402.2	2	K.SQQSEVT
**1 1** 2.846 0.00 TYMP_IPI:IPI00292858.4	☐	1126	1/1	2	14/18	0.4039	0.4039	2.9602	SH3BGRL3_IPI:IPI00010402.2	2	R.VYSTSVT
**1 1** 2.833 0.00 IPI:IPI00383732.1_IPI:IPI00383732.1	☐	3449	1/1	2	16/20	0.2538	0.2538	3.0874	HSP90B1_IPI:IPI00027230.3		K.SILFVPTS
**1 1** 2.819 0.00 COPB2_IPI:IPI00220219.6	☐	2671	1/7	2	11/16	0.1003	0.1003	2.0629	HSP90B1_IPI:IPI00027230.3		R.SGYLLPD
**1 1** 2.816 0.00 EEF1A2_IPI:IPI00014424.1	☐	3448	1/1	2	25/34	0.3165	0.3165	5.7174	PKM2_IPI:IPI00220644.8	5	K.GADFLV
**1 1** 2.812 0.00 APEH_IPI:IPI00337741.4	☐	3029	1/1	2	24/30	0.4022	0.4022	5.0664	ACTN4_IPI:IPI00013808.1	1	R.ETTDTD

Mass spectrometry for Band 3

The band indicating binding between nuclear extract of *ZNF423* variant LCLs treated with E2+4-OH-TAM and variant DNA probe in Figure 1B

Protein matches					☐	scanf	Rank_Sp	charge	Ions	dCn	dCn2	XCorr	Reference	Redu	Peptide
**7**	**7**	2.624	0.00	NCL_IPI:IPI00183526.6	☐	1710	1/1	2	18/22	0.3751	0.3751	3.2889	NCL_IPI:IPI00183526.6	4	K.GLSEDTTE
**5**	**5**	2.955	0.00	SFN_IPI:IPI00013890.2	☐	2853	1/1	3	27/76	0.2215	0.2215	3.1886	NCL_IPI:IPI00183526.6	4	K.GLSEDTTE
**4**	**4**	2.992	0.00	TXN_IPI:IPI00216298.6	☐	0920	1/3	2	10/12	0.1027	0.1027	1.8357	NCL_IPI:IPI00183526.6	4	K.GQNQDYR
**4**	**4**	2.859	0.00	NCL_IPI:IPI00444262.3	☐	1365	1/2	2	13/16	0.1527	0.1527	2.5683	NCL_IPI:IPI00183526.6	3	K.NSTWSGE
**3**	**3**	4.144	0.00	CALML3_IPI:IPI00216984.5	☐	1080	1/12	2	11/14	0.2257	0.2257	2.1711	NCL_IPI:IPI00183526.6	4	K.QGTEIDGR
**3**	**3**	3.772	0.00	PCNA_IPI:IPI00021700.3	☐	2184	1/37	2	10/12	0.0860	0.0860	2.2779	NCL_IPI:IPI00183526.6	4	R.LELQGPR.
**3**	**3**	3.420	0.00	YWHAG_IPI:IPI00220642.7	☐	2410	1/1	2	15/18	0.2396	0.2396	3.0367	NCL_IPI:IPI00183526.6	4	R.SISLYYTG
**3**	**3**	3.073	0.00	YWHAE_IPI:IPI00000816.1	☐	0504	1/23	2	11/14	0.2100	0.2100	1.8957	SFN_IPI:IPI00013890.2		K.EAGDAES
**3**	**3**	2.986	0.00	GAPDH_IPI:IPI00219018.7	☐	2167	1/19	3	21/52	0.2531	0.2531	3.4835	SFN_IPI:IPI00013890.2	1	K.SNEEGSEE
**3**	**3**	2.927	0.00	ANXA2P2_IPI:IPI00334627.3	☐	2343	1/1	3	28/92	0.1157	0.1157	3.9363	SFN_IPI:IPI00013890.2	1	R.DNLTLWT
**2**	**2**	3.795	0.00	ATP5B_IPI:IPI00303476.1	☐	1765	1/2	2	12/14	0.0574	0.0574	2.5416	SFN_IPI:IPI00013890.2	1	R.VLSSIEQK.
**2**	**2**	3.374	0.00	TUBA1C_IPI:IPI00166768.3	☐	2135	1/1	2	18/20	0.2283	0.2283	2.9159	SFN_IPI:IPI00013890.2	1	R.YLAEVAT
**2**	**2**	2.818	0.00	ACTA2_IPI:IPI00008603.1	☐	3093	1/2	2	16/20	0.2067	0.2067	3.2255	TXN_IPI:IPI00216298.6	1	K.EKLEATIN
**2**	**2**	2.355	0.00	TCEB2_IPI:IPI00026670.3	☐	2582	1/14	2	12/16	0.1192	0.1192	2.3216	TXN_IPI:IPI00216298.6	1	K.LEATINEL
**1**	**1**	5.804	0.00	MYH9_IPI:IPI00019502.3	☐	2725	1/1	2	20/24	0.3089	0.3089	3.9014	TXN_IPI:IPI00216298.6	1	K.TAFQEAL
**1**	**1**	5.345	0.00	YWHAZ_IPI:IPI00021263.3	☐	1916	1/2	2	12/16	0.3591	0.3591	2.5202	TXN_IPI:IPI00216298.6	1	K.VGEFSGA
**1**	**1**	5.139	0.00	ACTBL2_IPI:IPI00003269.1	☐	2405	1/13	2	14/18	0.1987	0.1987	3.0824	NCL_IPI:IPI00444262.3	3	K.EVFEDAA
**1**	**1**	5.006	0.00	ANXA2_IPI:IPI00418169.3	☐	2281	1/1	2	15/16	0.3755	0.3755	3.4358	NCL_IPI:IPI00444262.3	2	K.NDLAVVD
**1**	**1**	4.900	0.00	SSBP1_IPI:IPI00029744.1	☐	2552	1/1	2	15/16	0.2995	0.2995	2.9782	NCL_IPI:IPI00444262.3	2	K.TGISDVFA
**1**	**1**	4.275	0.00	NACA_IPI:IPI00023748.3	☐	1086	1/90	2	9/12	0.0511	0.0511	1.9396	NCL_IPI:IPI00444262.3	3	K.VTQDELK.
**1**	**1**	4.091	0.00	CALML5_IPI:IPI00021536.2	☐	2400	1/1	2	15/20	0.1353	0.1353	3.0156	CALML3_IPI:IPI00216984.5		K.LSDEEVD
**1**	**1**	3.902	0.00	HNRNPAB_IPI:IPI00334587.1	☐	2309	1/1	2	24/32	0.4545	0.4545	5.7947	CALML3_IPI:IPI00216984.5		R.AADTDGD
**1**	**1**	3.773	0.00	HSPB1_IPI:IPI00025512.2	☐	2213	1/1	2	17/22	0.3187	0.3187	3.6209	CALML3_IPI:IPI00216984.5		R.SLGQNPTE
**1**	**1**	3.755	0.00	EIF6_IPI:IPI00010105.1	☐	2172	1/1	2	21/24	0.3687	0.3687	3.9321	PCNA_IPI:IPI00021700.3		K.FSASGELG
**1**	**1**	3.716	0.00	HMGB3L1_IPI:IPI00006437.2	☐	0821	1/7	2	10/12	0.1725	0.1725	1.9883	PCNA_IPI:IPI00021700.3		K.VSDYEM*
**1**	**1**	3.402	0.00	HSP90AA1_IPI:IPI00382470.3	☐	2841	1/1	2	23/36	0.4484	0.4484	5.3956	PCNA_IPI:IPI00021700.3		R.AEDNADT
**1**	**1**	3.252	0.00	HIST1H4J_IPI:IPI00453473.6	☐	2089	1/1	2	22/26	0.3623	0.3623	4.6718	YWHAG_IPI:IPI00220642.7	1	K.NVTELNE
**1**	**1**	3.241	0.00	LMNA_IPI:IPI00021405.3	☐	0842	1/2	2	15/16	0.2387	0.2387	2.6324	YWHAG_IPI:IPI00220642.7	1	R.ATVVESSE
**1**	**1**	3.188	0.00	TUBB_IPI:IPI00011654.2	☐	2280	1/1	2	16/18	0.3189	0.3189	2.9567	YWHAG_IPI:IPI00220642.7		R.YLAEVAT
**1**	**1**	3.100	0.00	A2ML1_IPI:IPI00419215.5	☐	1968	1/1	2	16/20	0.2896	0.2896	3.1061	YWHAE_IPI:IPI00000816.1	1	K.EAAENSL
**1**	**1**	3.066	0.00	IPI:IPI00383732.1_IPI:IPI00383732.1	☐	1274	1/1	2	17/20	0.2998	0.2998	3.8137	YWHAE_IPI:IPI00000816.1		K.EALQDVE
**1**	**1**	3.028	0.00	TUBB2C_IPI:IPI00007752.1	☐	1034	1/2	2	11/12	0.0809	0.0809	2.2988	YWHAE_IPI:IPI00000816.1	19	K.LAEQAER.
**1**	**1**	3.020	0.00	HSPA8_IPI:IPI00003865.1	☐	2418	1/1	2	12/14	0.1157	0.1157	2.5300	GAPDH_IPI:IPI00219018.7	3	K.VGVNGFG
**1**	**1**	2.944	0.00	EEF1A2_IPI:IPI00014424.1	☐	2671	1/25	2	16/28	0.2474	0.2474	3.1951	GAPDH_IPI:IPI00219018.7	5	R.GALQNIIP
**1**	**1**	2.873	0.00	ACTB_IPI:IPI00021439.1	☐	2964	1/1	3	25/72	0.3281	0.3281	3.2320	GAPDH_IPI:IPI00219018.7	5	R.GALQNIIP
**1**	**1**	2.855	0.00	P4HB_IPI:IPI00010796.1	☐	1918	1/1	2	15/16	0.3310	0.3310	2.4304	ANXA2P2_IPI:IPI00334627.3	5	K.AYTNFDA
**1**	**1**	2.814	0.00	IGLC1_IPI:IPI00154742.6	☐	2451	1/7	2	14/18	0.1372	0.1372	2.9165	ANXA2P2_IPI:IPI00334627.3	5	R.DALNIETA

Venn diagram for proteins identified by mass spectrometry

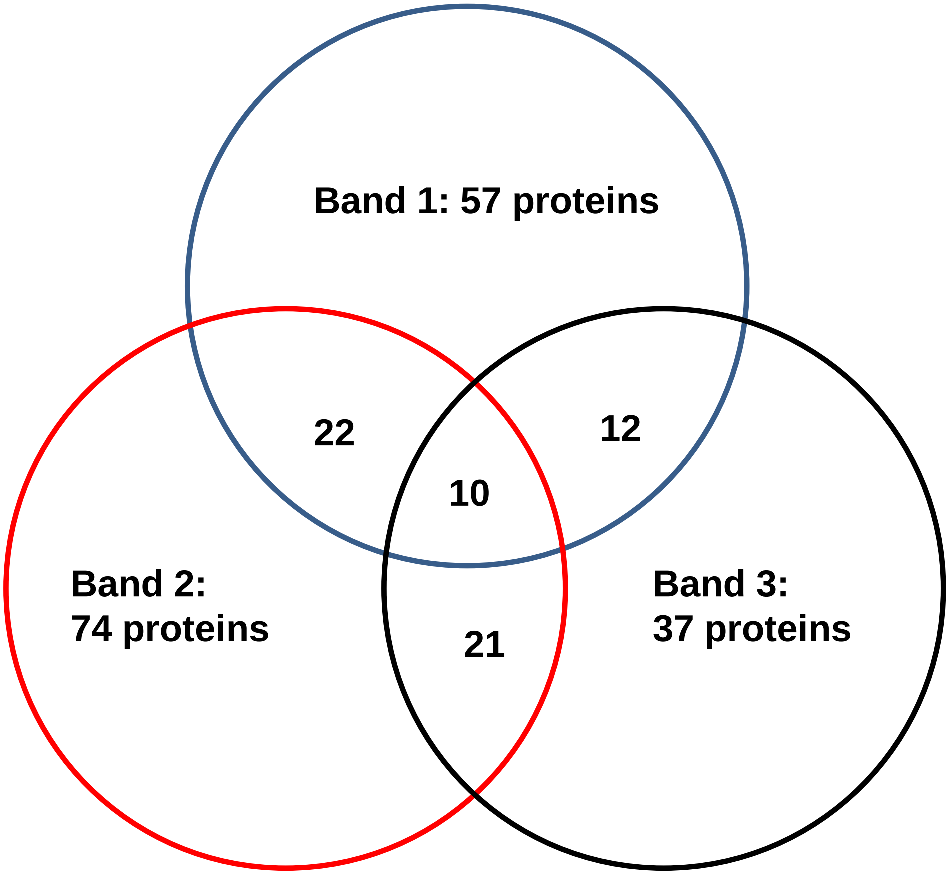

Supplement: Supplementary file 1 — Presents the MS results. (PDF 8620 kb) [file 13058_2017_890_MOESM1_ESM.pdf]
