# Supplementary material for: Calmodulin-like protein 3 is an estrogen receptor alpha coregulator for gene expression and drug response in a SNP, estrogen, and SERM-dependent fashion
Source: Breast Cancer Res. 2017 Aug 18;19:95. doi: 10.1186/s13058-017-0890-x (PMC5562991; doi:10.1186/s13058-017-0890-x)
Supplement: Supplementary file 2 — showing supplementary results. (PDF 10332 kb) [file 13058_2017_890_MOESM2_ESM.pdf]

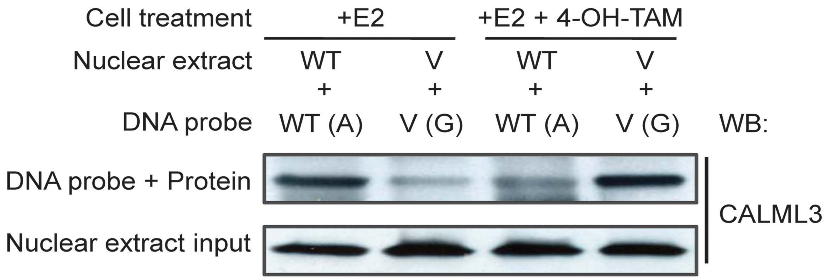

Figure S1

Detection of the candidate protein CALML3 after EMSA assay. DNA probes containing either WT or variant *ZNF423* rs9940645 SNP sequences were incubated with nuclear protein extract from LCLs carrying WT or variant SNP genotypes that had been treated with E2 ± 4-OH-TAM. CALML3 protein was detected with the specific CALML3 antibody using the EMSA membrane.

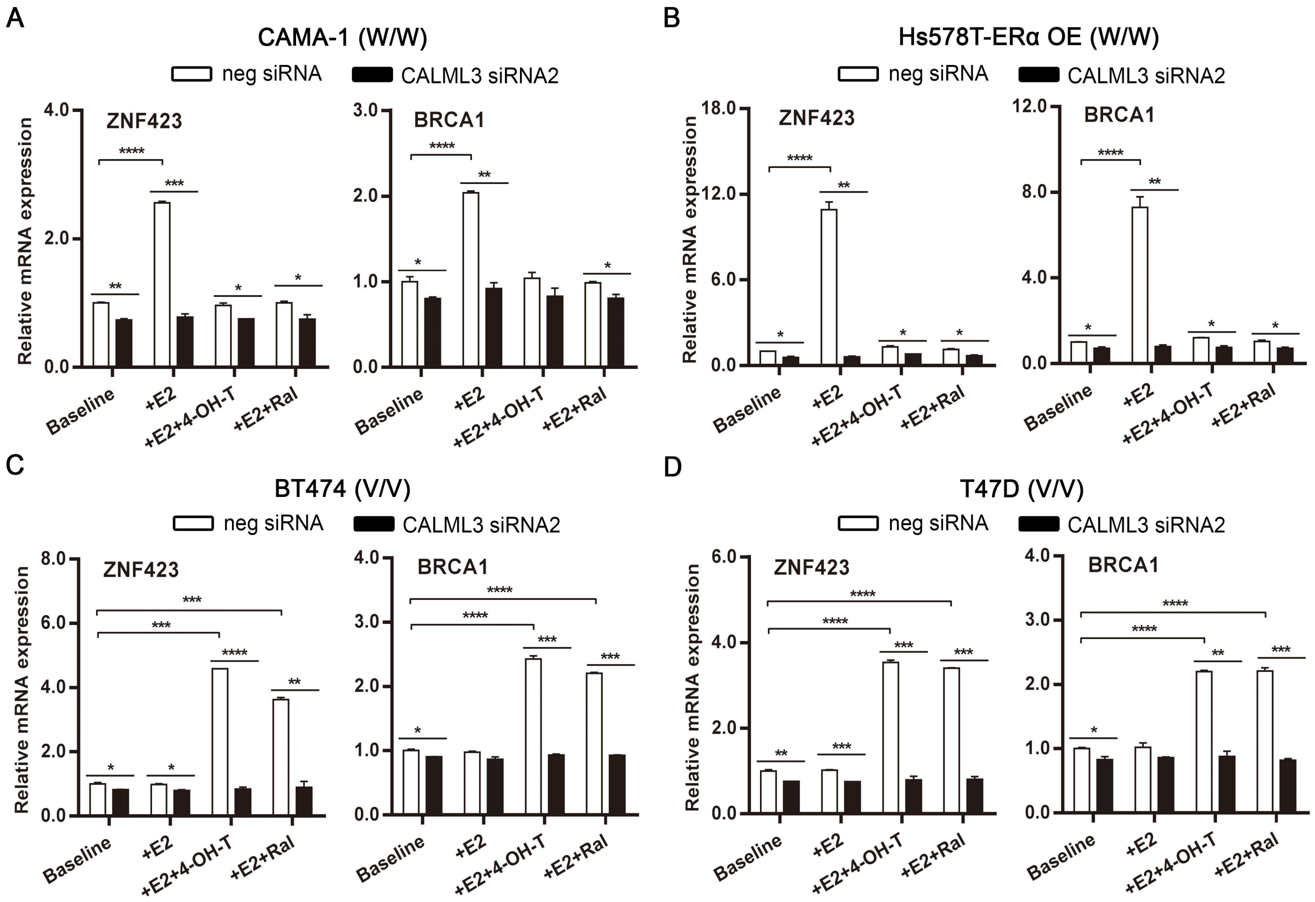

Figure S2

The expression of ZNF423 and BRCA1 determined by qRT-PCR in a panel of breast cancer cells after knocking down CALML3 using a second siRNA. The ZNF423 and BRCA1 mRNA expression levels relative to actin are shown as means of three independent experiments (± SEM), and comparisons were performed using a two-tailed Student's t-test, *p <0.05, **p <0.01, ***p < 0.001, ****p <0.0001.

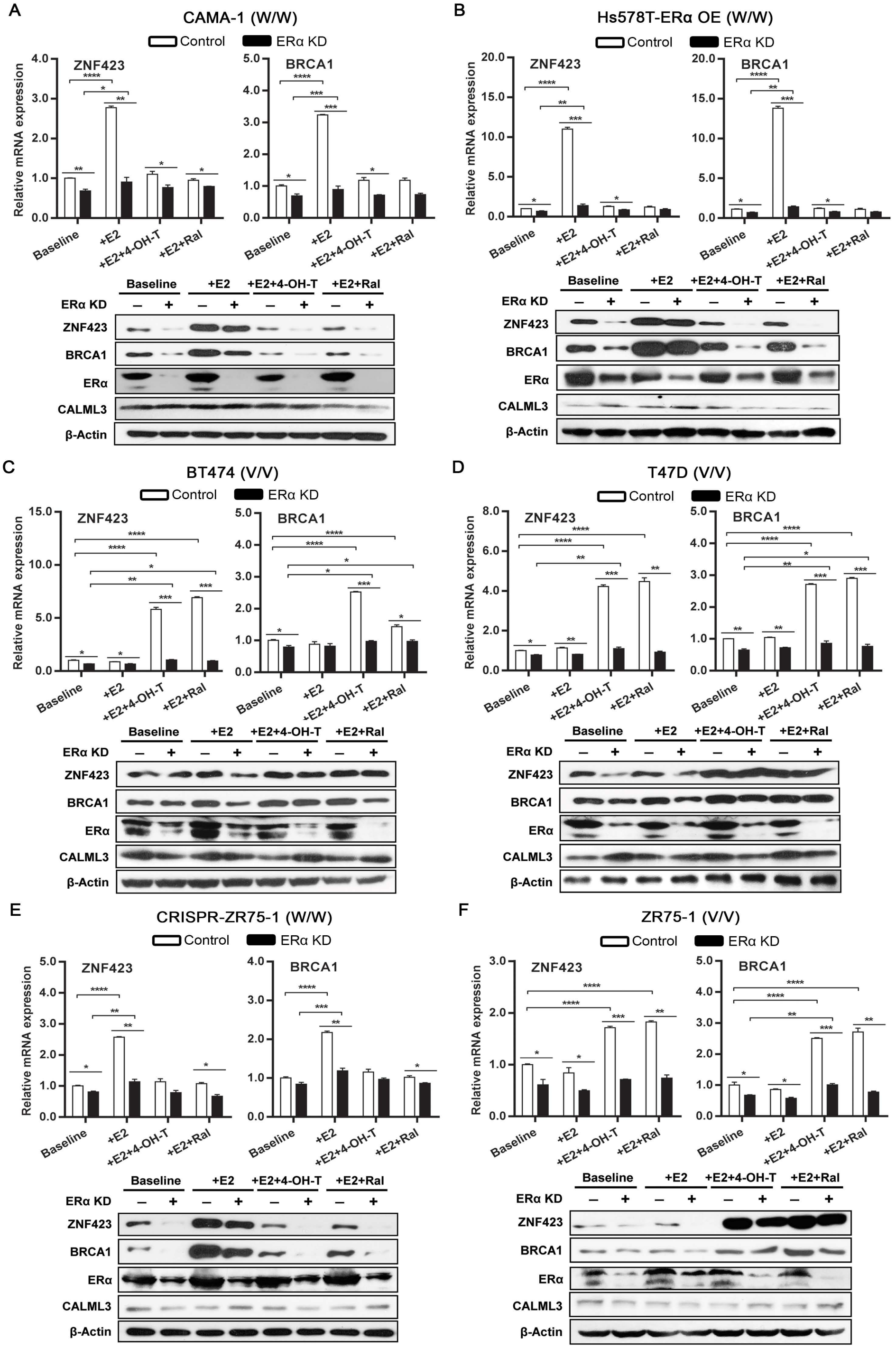

Figure S3

The expression of ZNF423 and BRCA1 determined by qRT-PCR in a panel of breast cancer cells including CRISPR-ZR75-1 cells (WT) and ZR75-1 cells (Variant) after knocking down ERα. Relative expression levels are shown as means of three independent experiments (± SEM), and comparison were made using a two-tailed Student's t-test, *p <0.05, **p <0.01, ***p < 0.001, ****p <0.0001. Protein levels were determined by Western Blot.

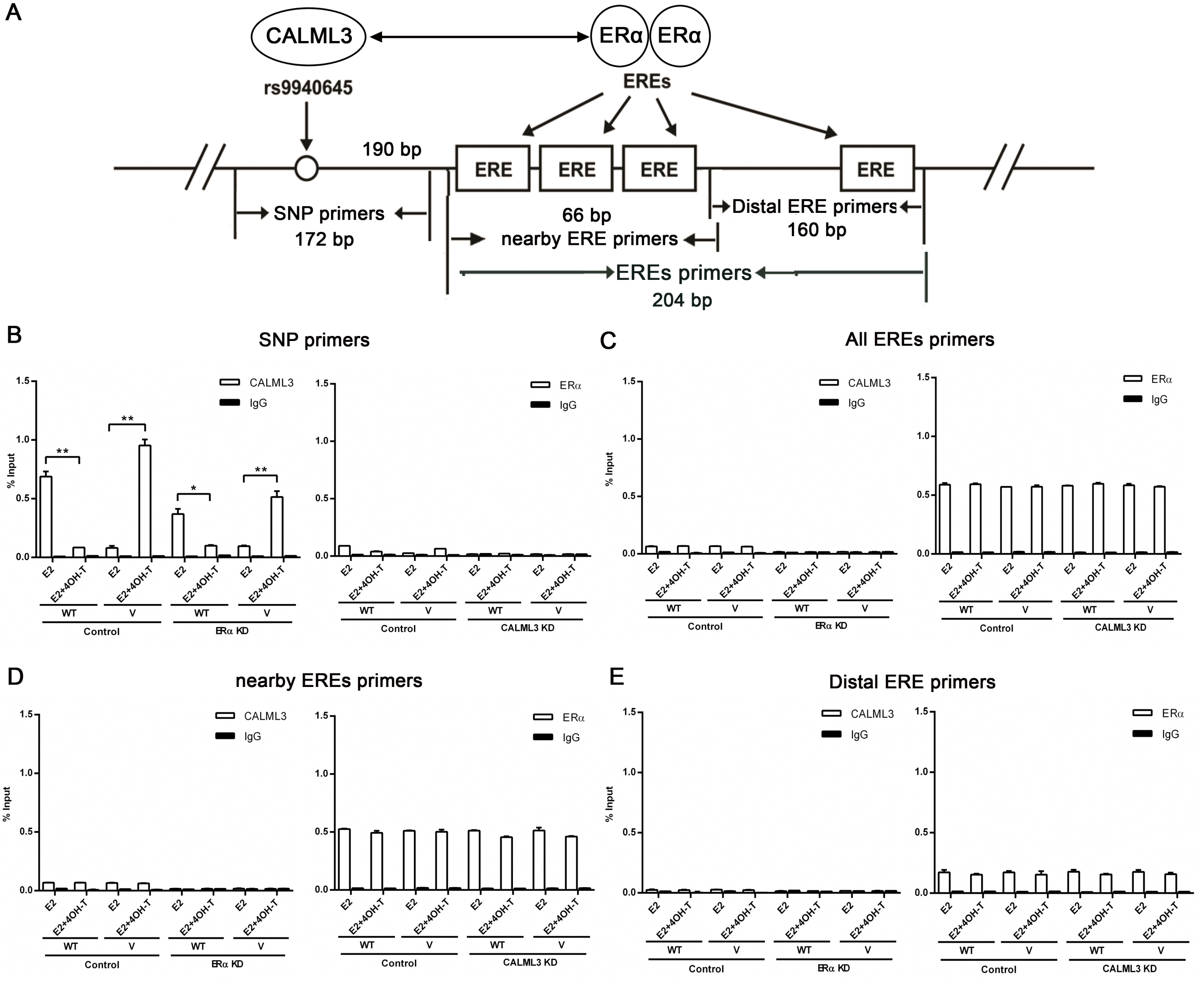

Figure S4

SNP-dependent regulation of CALML3 and ERα. ChIP-qPCR assays using CALML3 antibody after knocking down ERα, or using ERα antibody after knocking down CALML3 in CRISPR-ZR75-1 cells (WT) and ZR75-1 cells (Variant) treated with E2 ± 4-OH-TAM/raloxifen. **(A)** The locations of primers used to perform the ChIP-qPCR are shown. **(B)** Amplicons amplified the region containing only the SNP. **(C)** Amplicons amplified the region containing all the EREs. **(D)** Amplicons amplified the region containing nearby EREs. **(E)** Amplicons amplified the region containing only the distal ERE. The enrichment of DNA fragments (three independent experiments, mean ± SEM) between different treatments were compared using two-tailed Student's t-test, *p <0.05, **p <0.01, ***p < 0.001.

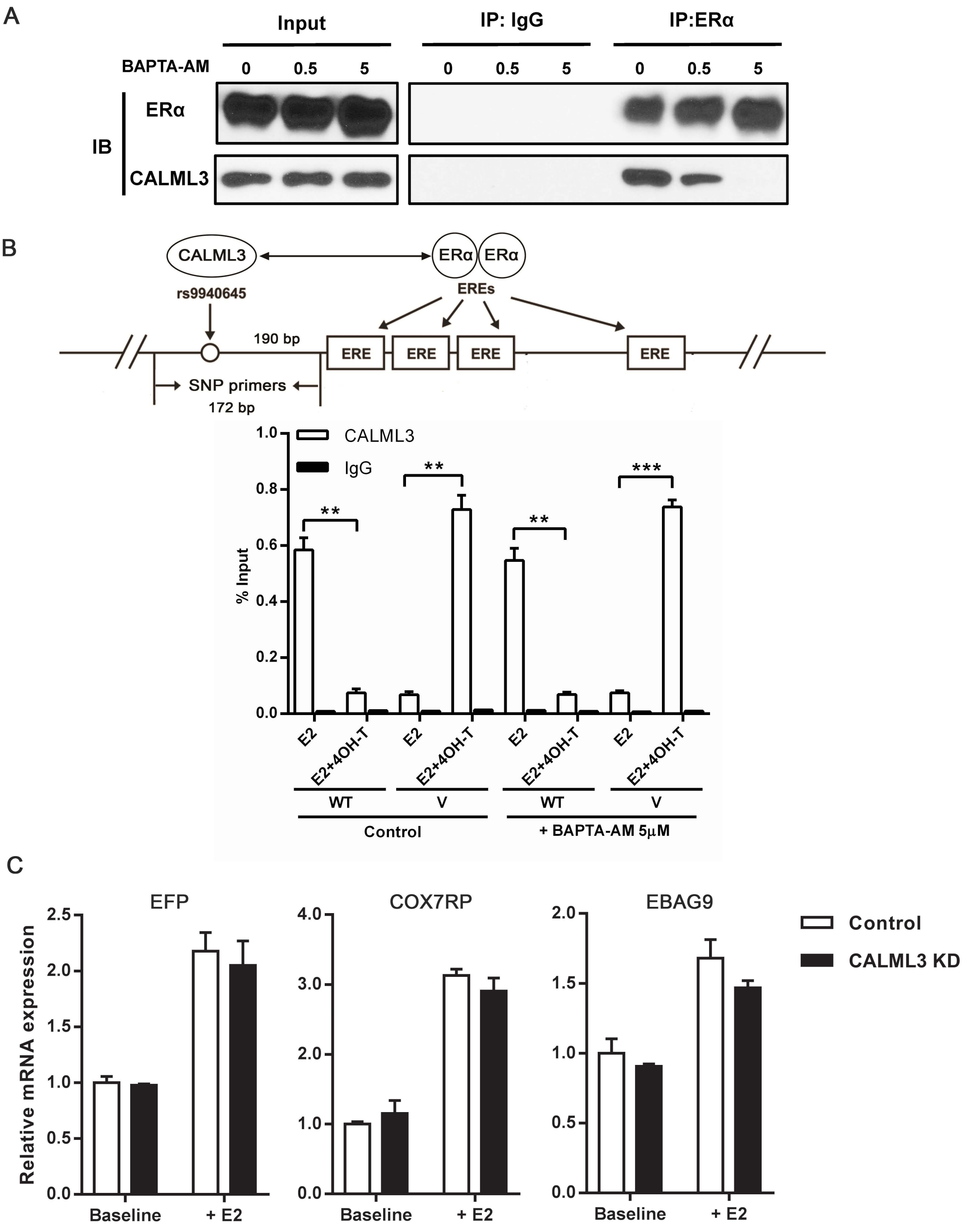

Figure S5

CALML3 binding to the ZNF423 SNP is calcium dependent but its interaction with ER is calcium independent. **(A)** CALML3-ER binding decreased significantly after calcium level was reduced with calcium chelator, BAPTA-AM at the concentration of 0.5 and 5 μM. **(B)** The binding between CALML3 and *ZNF423* SNP was independent on calcium. **(C)** Expression of canonical ER targeted genes did not change after down regulating CALML3 in the presence of E2 treatment.

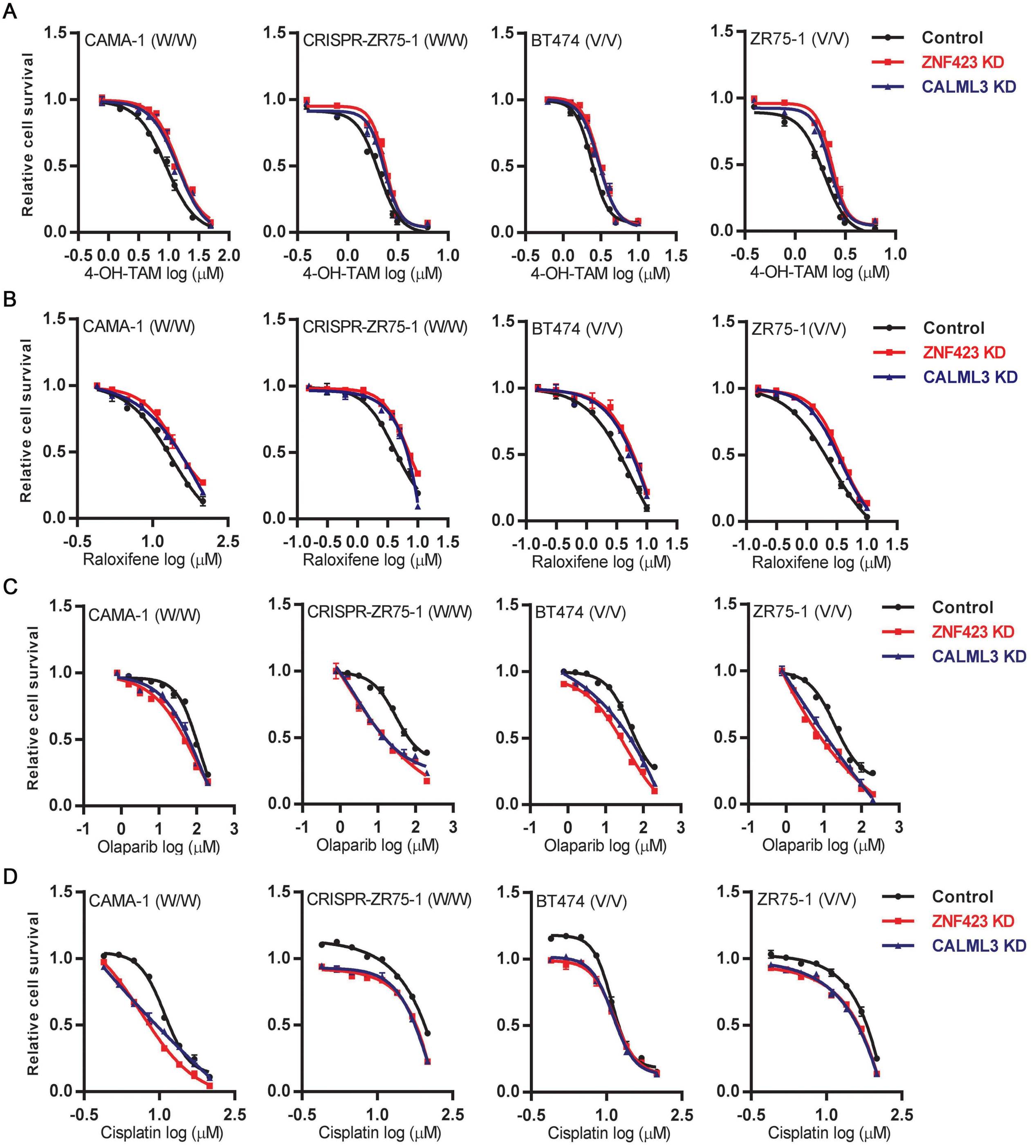

Figure S6

Drug cytotoxicity assays performed with a panel of breast cancer cells after the down regulation of ZNF423 or CALML3. Two representative breast cancer cell lines with the same *ZNF423* SNP genotype were selected and treated with a series of drug concentrations together with 0.01 nM E2 for 3 days, and cell viability was determined by MTS assay. **(A)** 4-OH-TAM. **(B)** Raloxifene. **(C)** Olaparib. **(D)** Cisplatin.

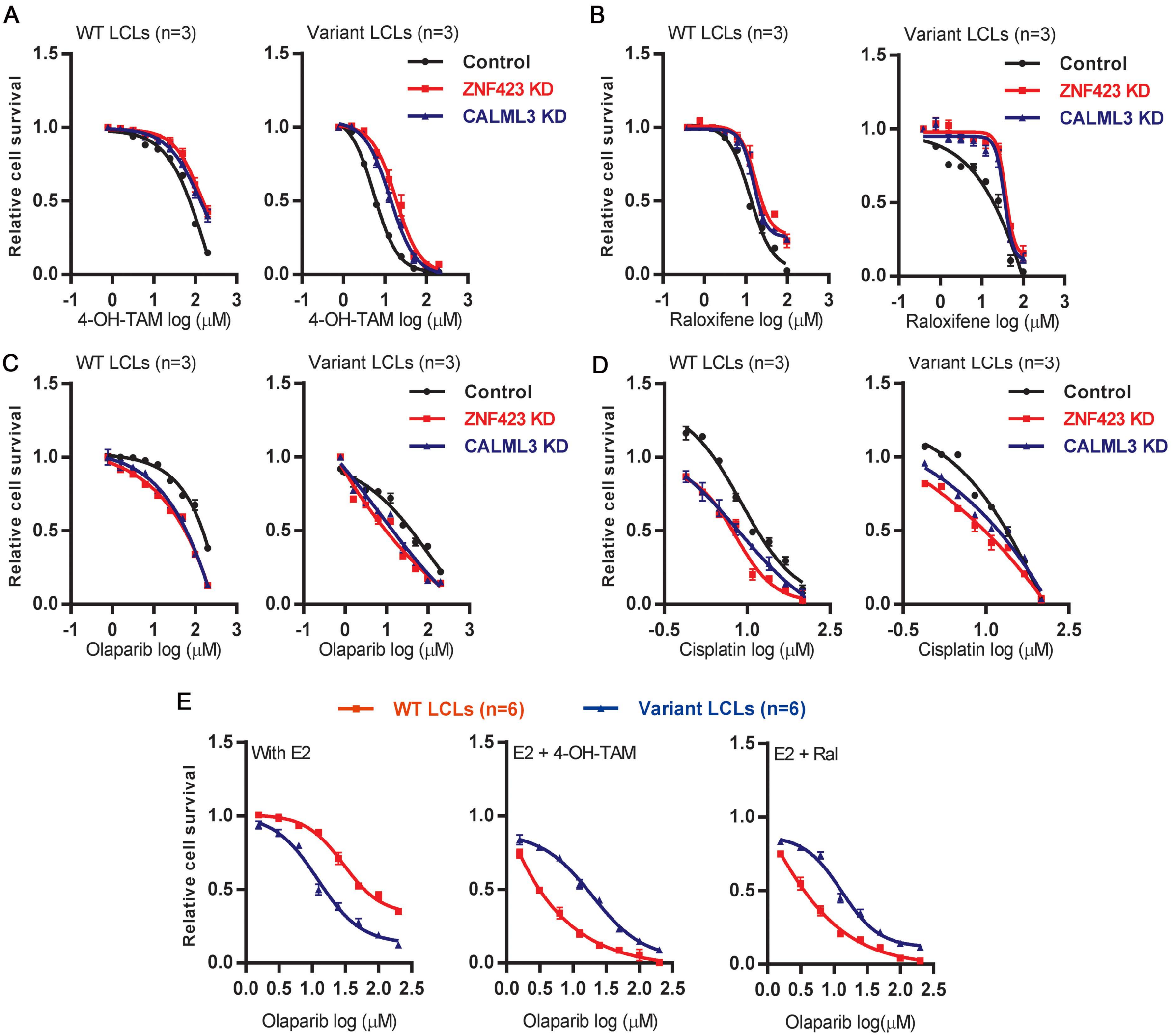

Figure S7

Drug cytotoxicity assays performed with a panel of LCLs with WT or variant *ZNF423* SNP genotypes treated with 4-OH-TAM, raloxifene, olaparib, and cisplatin after the down regulation of ZNF423 or CALML3, or treated with olaparib alone or in combination with 4-OH-TAM or raloxifene. The drug treatments were in the presence of 0.01 nM E2 and the viability of cells after treatments for 3 days were determined by MTS assay. **(A)** 4-OH-TAM. **(B)** Raloxifene. **(C)** Olaparib. **(D)** Cisplatin. **(E)** Olaparib ± 4-OH-TAM/raloxifene.

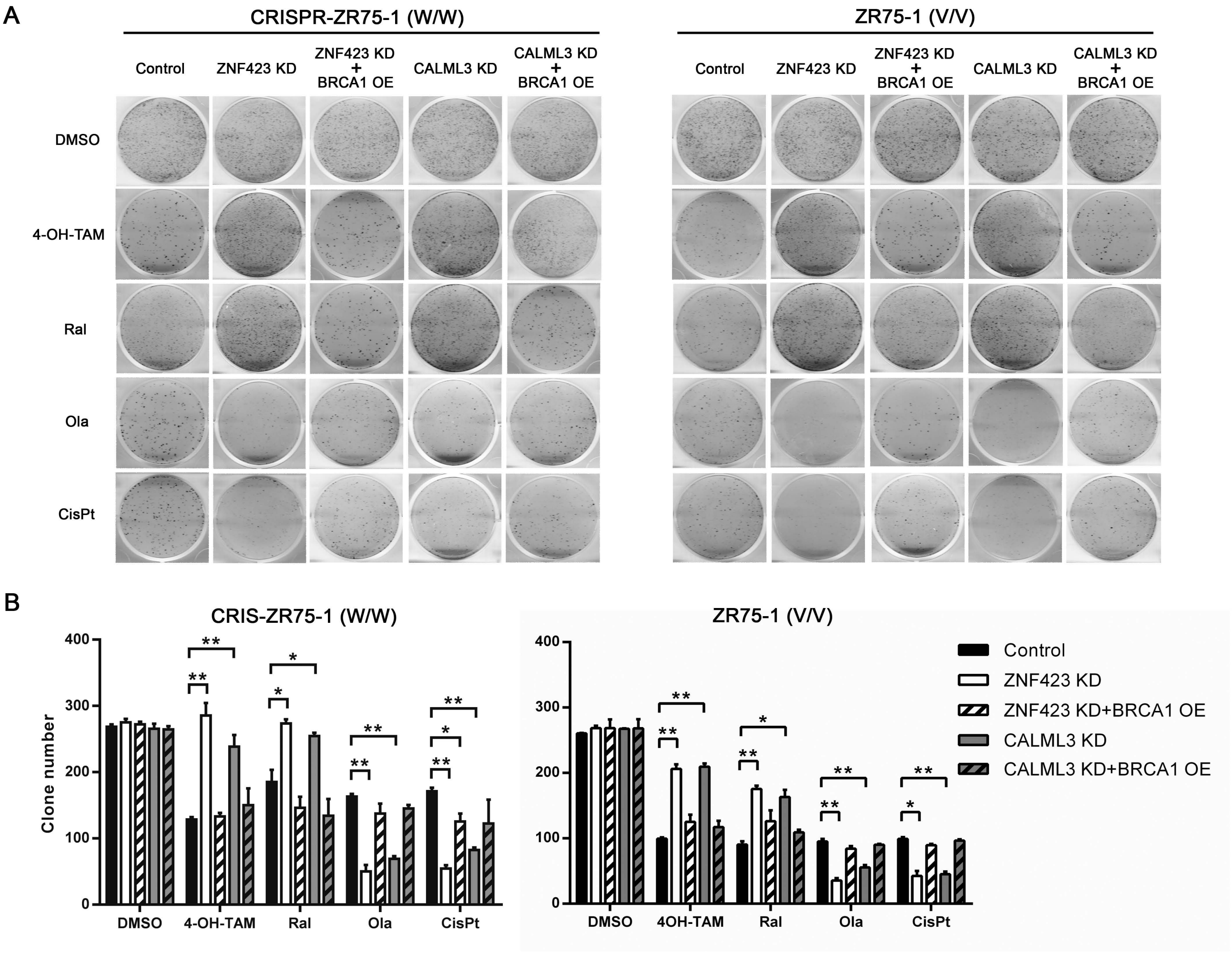

Figure S8

Colony formation in ZR75-1 cells with different ZNF423 SNP genotypes with drug treatments. **(A)** Representative clones are shown for ZR75-1 and CRISPR-ZR75-1 cells treated with the indicated drugs for 4 weeks. **(B)** Clone number was counted in each well, shown here as three independent experiments, Mean ± SEM. Comparisons with the control in each treatment were performed by two-tailed Student's t-test, **p* <0.05, ***p* <0.01.
